# TFAP4 exacerbates pathological cardiac fibrosis by modulating mechanotransduction

**DOI:** 10.1016/j.cellin.2025.100256

**Published:** 2025-06-02

**Authors:** Jie Liu, Jingjing Feng, Jingxuan Zhao, Xiangjie Kong, Zhangyi Yu, Yuanru Huang, Zechun He, Mengxin Liu, Zheng Liu, Zhibing Lu, Li Wang

**Affiliations:** a Department of Cardiology, Zhongnan Hospital of Wuhan University, Medical Research Institute, Frontier Science Center for Immunology and Metabolism, Wuhan University, Wuhan 430071, Hubei, China; b Institute of Myocardial Injury and Repair, Wuhan University, Wuhan 430071, Hubei, China; c Hubei Provincial Clinical Research Center for Cardiovascular Intervention, Wuhan 430071, Hubei, China; d The Institute for Advanced Studies, TaiKang Center for Life and Medical Sciences, Hubei Key Laboratory of Cell Homeostasis, College of Life Sciences, Wuhan University, Wuhan 430072, Hubei , China

**Keywords:** Cardiac fibrosis, Fibroblast activation, Mechanotransduction, *TFAP4*

## Abstract

Cardiac fibroblast (CF) differentiation into myofibroblasts is a crucial driver of cardiac fibrosis, leading to extensive extracellular matrix (ECM) deposition that increases myocardial stiffness and eventually impairs heart function. Mechanotransduction has merged as a key regulator of CF activation and the fibrotic response post-myocardial infarction (MI). However, the molecular mechanisms linking CF activation to mechanical cues within the injured myocardium remain poorly understood. Here we identified transcription factor TFAP4 as a central regulator of fibrosis in both human and murine models. TFAP4 overexpression enhances CF proliferation, ECM protein expression, and myofibroblast differentiation. Notably, TFAP4 directly activates expression of mechanosensors including Itga11 and Piezo2, which are essential for transmitting mechanical signals that promote CF activation and fibrosis. Silencing *Itga11* and *Piezo2* reverses the pro-fibrotic effects of TFAP4, while TFAP4 downregulation in vivo reduces fibrosis and improves cardiac function post-MI. These findings identify TFAP4 as a pivotal link between mechanotransduction and fibrosis, suggesting it as a potential therapeutic target to mitigate fibrosis and enhance cardiac recovery following MI.

## Highlights

• Functional screening identifies TFAP4 as a key regulator of cardiac fibroblast activation.
• TFAP4 drives cardiac fibrosis by activating the mechanotransduction pathway.
• TFAP4 directly binds to the promoters of Itga11 and Piezo2, upregulating their expression to enhance fibrosis.

## Graphical abstract

**Figure:**
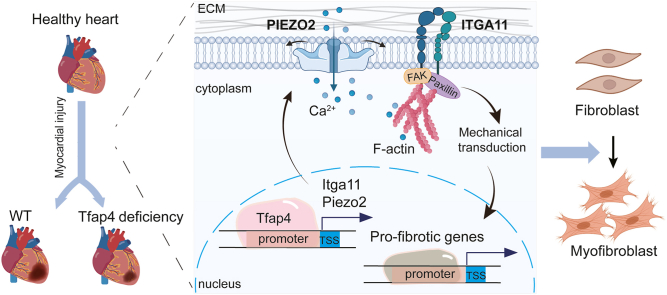

## 1 Introduction

Despite significant advancements in cardiovascular treatments, myocardial infarction (MI) caused by blockage of the coronary arteries continues to be a leading cause of morbidity and mortality worldwide(Murray & Lopez, 2013). Following MI, cardiac fibroblasts (CFs) undergo a transformation into myofibroblasts, which is characterized by rapid proliferation and migration to the injury site. These myofibroblasts produce a collagen-rich extracellular matrix (ECM) that forms scar tissue and preserves cardiac structure and function(Daseke et al., 2020; Ivey & Tallquist, 2016). However, excessive ECM deposition ultimately leads to increased myocardial stiffness, reduced tissue elasticity and impaired cardiac function(Pesce et al., 2023a). Therefore, a deeper understanding of the molecular mechanisms driving fibroblast activation in response to MI is critical for developing novel therapeutic strategies to restore heart function.

Mechanical forces serve as fundamental regulators of tissue homeostasis, especially in mechanically active organs like the heart(Ladoux & Mège, 2017; Romani et al., 2021a). After MI, the cardiac mechanical properties shift due to altered pressure and tissue stiffness, creating a pro-fibrotic environment. Cardiac fibroblasts sense these mechanical signals through a variety of mechanosensors including integrins and ion channels such as Piezo1 and Piezo2(Coste et al., 2010). These mechanical cues are converted into biochemical signals that activate intracellular cascades transmitted via the cytoskeleton and various kinases including mitogen-activated protein kinase (MAPK) p38, extracellular signal-regulated protein kinases 1 and 2 (ERK1/2) and protein kinase B (PKB/Akt). These pathways ultimately lead to the transcriptional activation of fibrotic gene expression and ECM production. While the transcription factors (TFs) downstream of mechanotransduction such as YAP/TAZ(Mia et al., 2022) and SMADs(Khalil et al., 2017) are well documented, the TFs that govern the expression of mechanotransduction pathways remain poorly understood.

Through dual-omics approaches, our previous work identified transcription factor activator protein 4 (TFAP4 or AP4) as a key TF enriched in cardiac fibroblasts(Wang et al., 2022b). TFAP4 is a member of the basic helix-loop-helix (bHLH) family and binds to conserved E-box sequence (CAGCTG) via homodimerization(Mermod et al., 1988). TFAP4 has been shown to play critical roles in modulating transcriptional networks related to cellular differentiation, proliferation, metastasis and other essential biological functions in cancer. In tumorigenesis, TFAP4 regulates genes involved in cellular senescence, such as p16 and p21(Jackstadt, Jung, & Hermeking, 2013), as well as genes like SNAIL and CDH1 associated with epithelial-mesenchymal transition (EMT)(Jackstadt, Röh, et al., 2013), and those in the Wnt/β-catenin signaling pathway, including DVL1 and LEF1(Song et al., 2018). Overexpression of TFAP4 is linked to multiple human malignancies including gastric and colorectal cancers, suggesting its oncogenic potential(Jackstadt, Röh, et al., 2013; Liu et al., 2012). However, its role in fibroblasts during cardiac remodeling was largely unexplored.

In this study, we identified Tfap4 as a central regulator of fibrosis in both human and murine models. We observed that Tfap4 overexpression enhances CF proliferation, ECM deposition, and the transition of CFs to a myofibroblast. In particular, Tfap4 directly activates expression of mechanosensors including Itga11 and Piezo2, which are essential for transmitting mechanical signals that promote CF activation and fibrosis. Silencing these mechanosensors reverses the pro-fibrotic effects of Tfap4, while in vivo *Tfap4* knockout reduces fibrosis and improves cardiac function post-MI. These findings reveal Tfap4 as a pivotal link between mechanotransduction and fibrosis, suggesting it as a potential therapeutic target to limit fibrosis and enhance recovery following MI.

## 2 Results

### 2.1 Identification of Tfap4 as a critical regulator for cardiac fibroblast activation

**Fig. 1:**
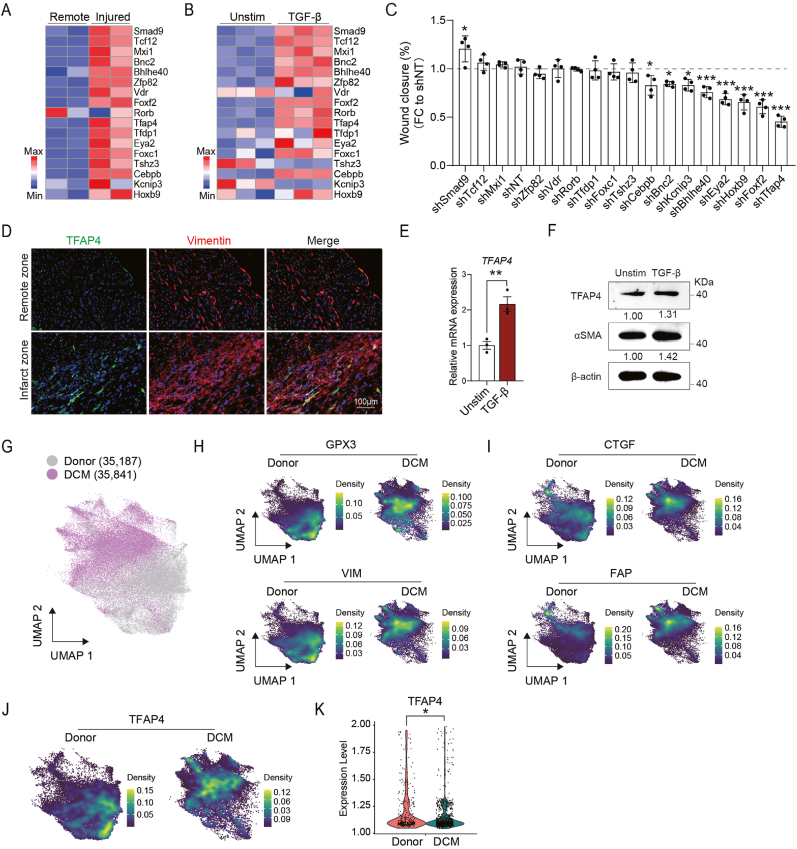
**Identification of Tfap4 as a crucial transcription factor.** (A) Heatmap showing RT-qPCR results evaluating the expression of candidate transcription factors in injured and remote regions of myocardium at 4 weeks post MI (*n* = 2, biological replicates). (B) Heatmap showing RT-qPCR results evaluating the expression of candidate transcription factors in CFs after TGF-β treatment for 24 h (*n* = 3, technical replicates). (C) Quantification of the wound healing rate 24 h after a scratch in CFs subjected to transduction of lentiviral shRNAs targeting indicated transcription factors. Lentiviral non-targeting shRNA (shNT) was used as control (*n* = 3, technical replicates). (D) Representative immunofluorescence staining images showing the expression of TFAP4 and its colocalization with Vimentin in the infarcted and remote region of myocardium at 4 weeks post MI. Scale bar, 100 μm. (E) RT-qPCR analysis showing the expression of *TFAP4* in unstimulated (Unstim) and TGF-β-treated human CFs (HCFs) (*n* = 3, technical replicates). (F) Representative Western blot and quantification showing TFAP4 and *α*-SMA protein expression in HCFs with or without TGF-β treatment. (G) Unsupervised clustering of fibroblast population from healthy donor (grey) and DCM (purple) hearts. (H) UMAP plot showing the expression of shared cardiac markers *GPX3* and *VIM* in donor and DCM group. (I) UMAP plot showing the expression of *CTGF*, *FAP* in donor and DCM group. (J) UMAP plot showing the expression of *TFAP4* in donor and DCM group. (K) Violin plot showing the expression level of TFAP4 in the TFAP4^+^ FBs from donor and DCM groups. All experiments were repeated at least three times. Data are presented as mean ± SEM. Groups were compared using two-tailed unpaired *t*-test (E) or one-way ANOVA with Tukey's multiple comparisons test (C). ^∗^*P* ​< ​0.05, ^∗∗^*P* ​< ​0.01, and ^∗∗∗^*P* < 0.001.

Our previous work, applying single-cell transcriptomic and epigenomic profiling of cardiac non-myocytes, identified a panel of TFs specifically enriched in CFs(Wang et al., 2022a). However, their role in CF function remained unclear. To identify novel TFs involved in cardiac fibrosis, we evaluated TF expression in response to injury and pro-fibrotic stimulations and ranked their ability to affect CF migration. Among the top 17 TFs identified from our dual-omics datasets(Wang et al., 2022a), we found that most of the TFs, including Tfap4, were upregulated in the infarcted zone compared to the remote zone in murine hearts post MI, as determined by reverse transcription-quantitative polymerase chain reaction (RT-qPCR) (Fig. 1(A) and Fig. S1A). To further evaluate the role of these TFs in fibroblast activation, we treated murine CFs with TGF-β, a canonical pro-fibrotic stimulus (Fig. 1(B)). After 24 h, CFs showed increased expression of fibrosis markers, including *Acta2* (which encodes α-SMA), *Postn*, *Ctgf*, *Wisp2*, *Col8a1* and *Fn1* (Fig. S1B). Interestingly, while a few TFs, such as *Tshz3* and *Kcnip3*, showed reduced expression, most TFs, including *Bnc2*(Bobowski-Gerard et al., 2022) and *Eya2*(Lee et al., 2009) known to promote fibrosis in liver or heart tissue, displayed increased expression upon TGF-β treatment (Fig. 1(B)).

Since fibroblast migration is crucial for wound healing post MI(Daseke et al., 2020), we next examined the effect of these TFs on CF migration. Using lentiviral shRNA-mediated knockdown, we systematically silenced each TF (Fig. S1C) and performed a scratch wound healing assay in primary CFs. We found that knockdown 8 out of the 17 TFs significantly impaired fibroblast migration, with *Tfap4* depletion causing the most profound reduction (Fig. 1(C) and Fig. S1D). Furthermore, immunostaining revealed that TFAP4 was abundantly expressed in Vimentin^+^ CFs within the infarct zone at 4 weeks post MI (Fig. 1(D)).

To investigate TFAP4 expression in human cardiac fibrosis, we first activated human cardiac fibroblasts (HCFs) with TGF-β and identified an increased *TFAP4* expression (Fig. 1(E)). Consistently, Western blot analyses confirmed the upregulation of TFAP4 and α-SMA, a myofibroblast marker, following TGF-β treatment (Fig. 1(F)). Next, we analyzed publicly available single cell transcriptomic datasets from healthy donor and dilated cardiomyopathy (DCM) hearts (Koenig et al., 2022). As reported (Koenig et al., 2022), we identified 14 distinct cell clusters, each of which was defined by canonical marker gene expression (Fig. S2A and B). TFAP4 was predominantly expressed in CFs, but also detectable at lower levels in other cells (Fig. S2C). Unsupervised clustering of fibroblasts recapitulated known fibroblast markers, including *GPX3* and *VIM* (shared between donor and DCM groups), and *CTGF* and *FAP* (upregulated in DCM fibroblasts) (Fig. 1(G)–(I)). Notably, TFAP4 was expressed in both donor and DCM fibroblasts, with higher levels in DCM fibroblasts (Fig. 1(J)–(K)). Together, these findings identify TFAP4 as a key regulator of CF activation and fibrosis, prompting further investigation into its mechanistic role in cardiac fibrosis.

### 2.2 Tfap4 promotes cardiac fibroblast activation

**Fig. 2:**
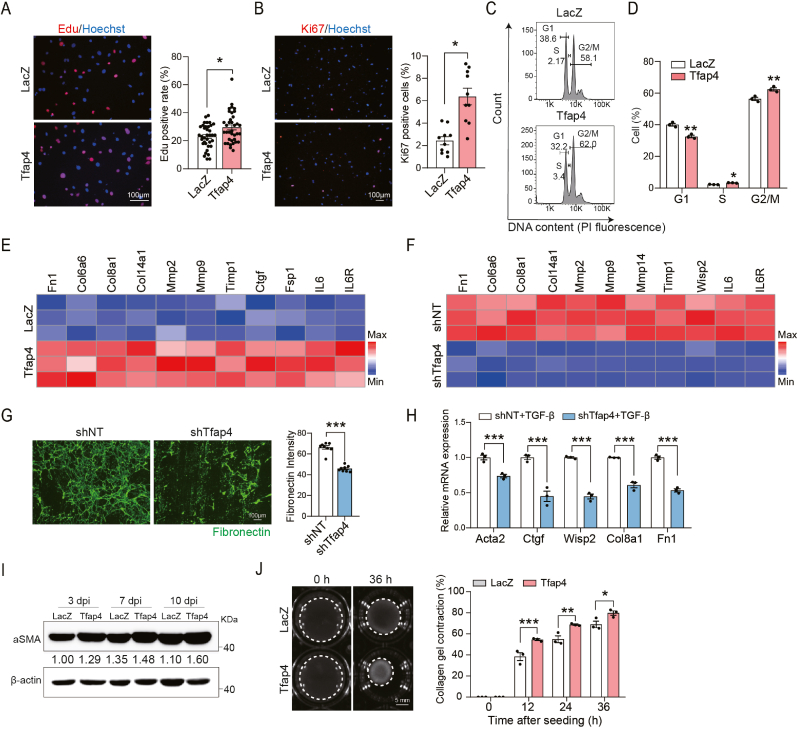
**Tfap4 positively regulates cardiac fibroblast activation.** (A)–(B) Representative ICC images and quantification of Edu^+^ (A) and Ki67^+^ (B) CFs that were transduced with lentiviruses harboring control LacZ fragment (control) or *Tfap4* (Tfap4-CFs) (*n* = 36 for (A); *n* = 10 for (B), biological replicates). Scale bar, 100 μm. (C)–(D). Representative flow cytometry plots (C) and quantification (D) of propidium iodide (PI) positive cells distributed in G0/G1, S or G2/M phases. CFs were transduced with lentiviral LacZ or *Tfap4* and subjected to PI staining at 72 h post-transduction (*n* = 3, biological replicates). (E) RT-qPCR analysis showing the expression of marker genes for activated fibroblasts in CFs at 5 days post lentiviral LacZ or *Tfap4* transduction (*n* = 3, technical replicates). (F) RT-qPCR analysis showing the expression of marker genes for activated fibroblasts in CFs at 5 days post lentiviral shNT or sh*Tfap4* transduction (*n* = 3, technical replicates). (G) Representative ICC images (left) and quantification (right) showing the deposition of fibronectin in CFs treated with shNT or sh*Tfap4* (*n* = 8, technical replicates). Scale bar, 100 μm. (H) RT-qPCR analysis showing the expression of activated fibroblast marker genes in TGF-β treated CFs that were transduced with shNT or sh*Tfap4* lentiviruses (*n* = 3, technical replicates). (I) Western blot and quantification showing *α*-SMA expression in LacZ-or Tfap4-CFs from day 3 to day 10 post-viral infection. (J) Representative bright-field images (left) and quantification (right) showing contraction area of floating collagen gels embedded with LacZ- or Tfap4-CFs at indicated time points (*n* = 3, biological replicates). Scale bar, 5 mm. All experiments were repeated at least three times. Data are presented as mean ± SEM. Groups were compared using two-tailed unpaired *t*-test ((A)-(B) and (G)) or two-way ANOVA with Sidak's multiple comparisons test ((D), (H) and (J)). ^∗^*P* ​< ​0.05, ^∗∗^*P* ​< ​0.01, and ^∗∗∗^*P* < 0.001.

To elucidate the role of Tfap4 in cardiac fibrosis, we combined cistromic and molecular analyses in primary CFs. We constructed a Tfap4 overexpression plasmid and measured the efficiency of *Tfap4* overexpression via RT-qPCR (Fig. S3A). We assessed the influence of Tfap4 on CF proliferation using 5-ethynyl-2′-deoxyuridine (Edu), a nucleotide analog that labels cells passing through S phase(Salic & Mitchison, 2008). Tfap4 knockdown significantly reduced the number of Edu^+^ CFs, while overexpression dramatically increased Edu^+^ CFs (Fig. 2(A) and Fig. S3B). Additional assays using Ki67 labeling and propidium iodide (PI) staining further supported that Tfap4 overexpression promotes CF proliferation, increasing the percentage of CFs in active phases of the cell cycle (Fig. 2(B)–(D) and ​Fig. S3 C and D). Consistently, sh*Tfap4* treatment significantly decreased the total cell number compared to the control group (Fig. S3E). These findings suggest that Tfap4 positively regulates CF proliferation.

Next, we sought to determine whether Tfap4 affects ECM protein composition by evaluating the expression of known regulators involved in pathological ECM remodeling. Our results showed that overexpression of *Tfap4* increased the expression of genes including matrix metalloproteinases (e.g., *Mmp2* and *Mmp9*) and their inhibitor (*Timp1*), collagens (e.g., *Col6a6*, *Col8a1* and *Col14a1*), and pro-fibrotic factors (e.g., *Ctgf* and *IL6*) (Goncalves et al., 2022; Takawale et al., 2017; Wang et al., 2024; Yokota et al., 2020)(Fig. 2(E)). Conversely, knockdown of *Tfap4* reduced the expression of these ECM genes (Fig. 2F). We also discovered that *Tfap4* knockdown attenuated mRNA and protein expression of fibronectin, a main component of ECM in response to cardiac damage(Wang et al., 2013) (Fig. 2(F)–(G)). Additionally, Tfap4 deficiency significantly decreased the expression of other fibrosis markers including *Acta2*, *Ctgf*, *Wisp2*, *Col8a1* and *Fn1* upon TGF-β treatment (Fig. 2(H)).

Conversion of quiescent fibroblasts to activated myofibroblasts is accompanied with α-SMA expression that is associated with scar contraction(Shinde et al., 2017). We found that forced expression of Tfap4 continuously increased α-SMA expression by approximately 1.5 ∼ 2-fold (Fig. 2(I)). In an in vitro collagen gel contraction assay, Tfap4 overexpression significantly enhanced contraction of fibroblast-populated collagen pads starting from 12 h and further increasing at 36 h after embedding the cells in the gel (Fig. 2(J)). In contrast, Tfap4 deficiency diminished the contractile ability of CFs seeded into collagen gels (Fig. S3F). Taken together, our results demonstrated that Tfap4 positively regulates fibroblast proliferation, activation and contractility, hallmarks of pathological fibrosis.

### 2.3 Overexpression of Tfap4 activates global transcription of fibrosis program

**Fig. 3:**
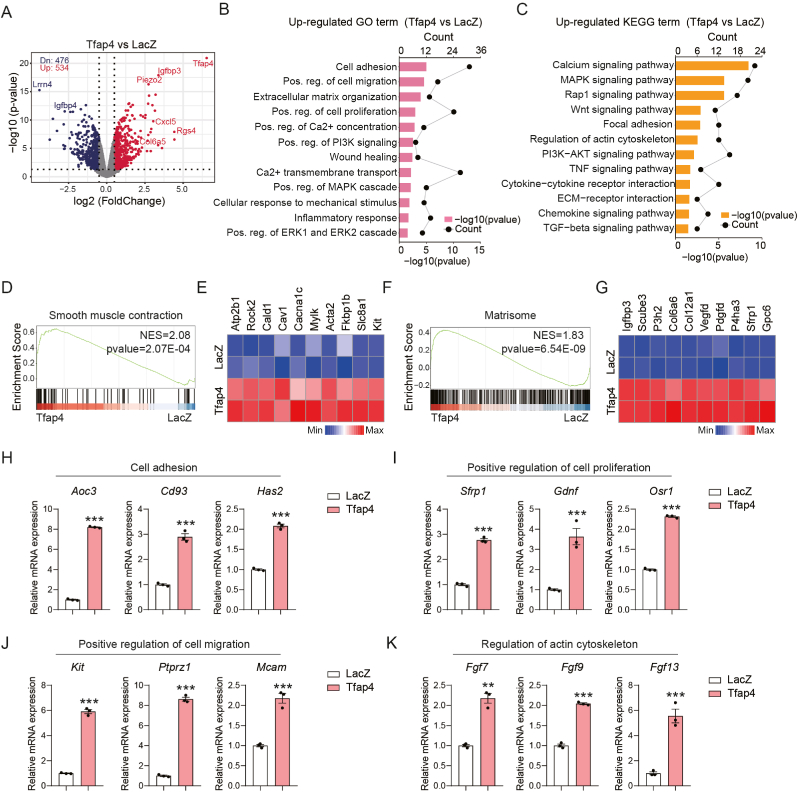
**Overexpression of Tfap4 activatespro-fibrotic gene network.** (A) Volcano plot depicting significantly enriched differentially expressed genes (DEGs) in Tfap4-CFs. DEGs were defined by *P* value < 0.05, |FC|≧1.5. (B) Gene ontology (GO) analysis showing the enriched biological processes associated with DEGs upregulated in Tfap4-CFs compared to LacZ control cells. Pos., positive; Reg., regulation. (C) KEGG enrichment analysis showing upregulated signaling pathways in Tfap4-CFs. (D) GSEA analysis showing the enrichment of the gene set associated with smooth muscle contraction in Tfap4-CFs compared to LacZ control cells. (E) Heatmap showing the expression of enriched representative genes correlating to (D). (F) GSEA analysis showing the enrichment of the gene set associated with matrisome in Tfap4-CFs compared to LacZ control cells. (G) Heatmap showing expression of enriched representative genes correlating to (F). (H) RT-qPCR analysis showing the expression of genes related to cell adhesion in control and Tfap4-CFs (*n* = 3, technical replicates). (I) RT-qPCR analysis showing the expression of genes related to positive regulation of cell proliferation in control and Tfap4-CFs (*n* = 3, technical replicates). (J) RT-qPCR analysis showing the expression of genes related to positive regulation of cell migration in control and Tfap4-CFs (*n* = 3, technical replicates). (K) RT-qPCR analysis showing the expression of genes related to regulation of actin cytoskeleton in control and Tfap4-CFs (*n* = 3, technical replicates). All experiments were repeated at least three times. Data are presented as mean ± SEM. Groups were compared using two-tailed unpaired *t*-test. ^∗∗^*P* ​< ​0.01, and ^∗∗∗^*P* < 0.001.

To elucidate the mechanisms by which TFAP4 promotes CF activation, we performed bulk RNA sequencing (RNA-seq) on CFs overexpressing *Tfap4* (Tfap4-CFs) or a LacZ control. We identified 534 upregulated and 476 downregulated transcripts in Tfap4-CFs (Fig. 3(A)). Among the upregulated genes, we observed increased expression of fibrosis-associated factors, including *Igfbp3*, *Rgs4*, and *Cxcl5*(Guo et al., 2021; Romani et al., 2021b; Sampson et al., 2013), while the anti-fibrotic gene *Igfbp4*(Su et al., 2019) was significantly downregulated. Gene ontology (GO) analysis revealed that *Tfap4* overexpression enriched pathways related to fibrosis, including cell adhesion, migration, proliferation, and ECM organization (Fig. 3(B)). Kyoto encyclopedia of genes and genomes (KEGG) analysis further identified activation of key fibrotic signaling pathways, including MAPK, Wnt, PI3K-AKT, and TGF-β (Fig. 3(C)). In contrast, downregulated genes were associated with inflammation and cell morphology (Fig. S4 A and B). Gene set enrichment analysis (GSEA) confirmed a significant enrichment of gene sets related to smooth muscle contraction and matrisome components in Tfap4-CFs (Fig. 3(D)–(G)).

To validate the transcriptomic findings, we selected top DEGs from each pathway for RT-qPCR analysis. *Tfap4* overexpression led to increased expression of adhesion-related genes (*Aoc3, Cd93, Has2*), proliferation markers (*Sfrp1, Gdnf, Osr1*), migration-associated genes (*Kit, Ptprz1, Mcam*), and regulators of actin cytoskeleton remodeling (*Fgf7, Fgf9, Fgf13*) (Fig. 3H–K). Collectively, these results demonstrate that *Tfap4* orchestrates a broad pro-fibrotic transcriptional program, establishing it as a key regulator of CF activation.

### 2.4 Tfap4 enhances mechanotransduction in cardiac fibroblasts

**Fig. 4:**
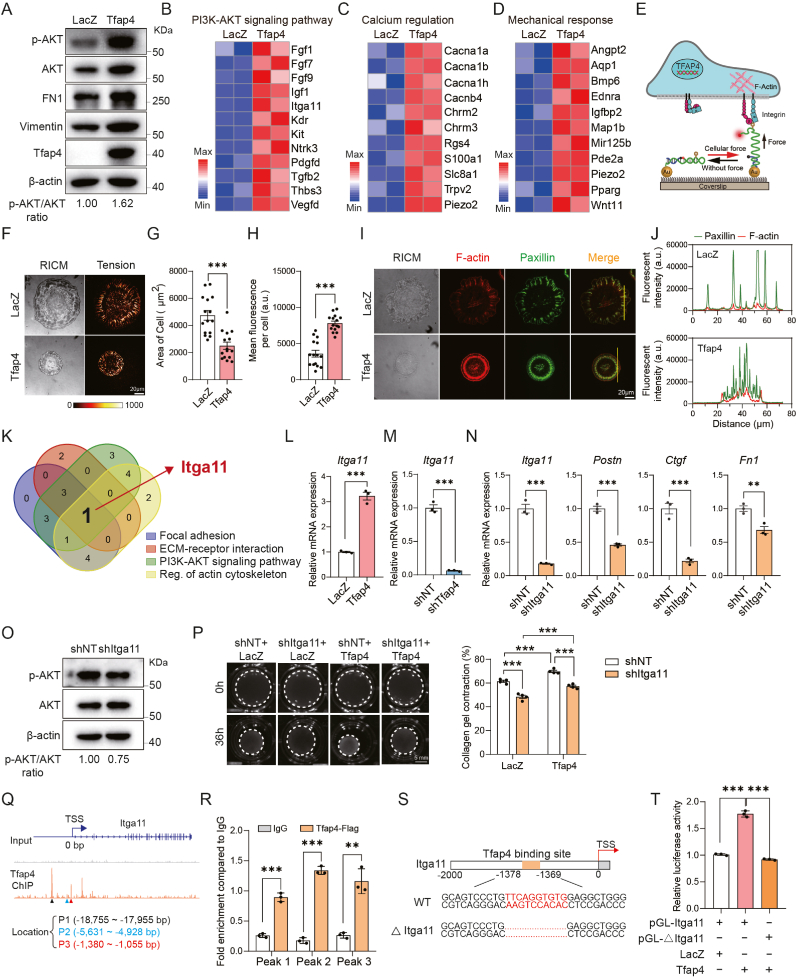
**Tfap4 activates mechanotransduction pathways including integrins to promote CF activation.** (A) Western blot and quantification showing Tfap4, Vimentin, FN1, AKT and pAKT expression in LacZ- or Tfap4-CFs. (B) Heatmap showing the expression of enriched genes correlating with PI3K-AKT signaling pathways. (C) Heatmap showing the expression of enriched genes correlating with calcium regulation. (D) Heatmap showing the expression of enriched genes correlating with mechanical response. (E) Schematic illustrating the mechanism of reversible shearing DNA-based tension probe to reflect the mechanic force of cell membrane exerted by the actin filaments and integrin. (F) Representative RICM and TIRF images of cell morphology and mechanic tension detected using DNA-based tension probe (17 pN) in LacZ or Tfap4-CFs. Scale bar, 20 μm. ((G)–(H**))** Quantification of spreading area (G) and tension intensity (H) of cells transduced with LacZ or *Tfap4* lentiviruses (*n* = 15, biological replicates). **(**I) Representative RICM and fluorescent images of Paxillin- and F-actin-tagged CFs treated with LacZ or *Tfap4* lentiviruses. Yellow lines in the fourth column indicate pixel regions used to generate fluorescence intensity profiles. Scale bar, 20 μm. (J) Quantification of Paxillin- and F-actin fluorescent intensity for cells with control LacZ or *Tfap4* overexpression. (K) The Venn diagram illustrates that Itga11 was identified as the common gene across mechanotransduction-related pathways (focal adhesion, ECM-receptor interaction, PI3K-AKT signaling pathway, regulation of actin cytoskeleton regulation) enriched among Tfap4-upregulated genes. (L) RT-qPCR analysis showing *Itga11* expression in control and Tfap4-CFs (*n* = 3, technical replicates). (**M**) RT-qPCR analysis showing *Itga11* expression in CFs treated with shNT or sh*Tfap4* (*n* = 3, technical replicates). (**N**) RT-qPCR analysis showing the expression of marker genes for activated fibroblasts in CFs at 5 days post lentiviral shNT or sh*Itga11* transduction (*n* = 3, technical replicates). (O) Representative Western blot and quantification showing AKT and pAKT protein expression in CFs treated with shNT or sh*Itga11.* (P) Representative bright-field images and quantification showing contraction area of floating collagen gels embedded with LacZ- or Tfap4-CFs treated with shNT or sh*Itga11* at indicated time points (*n* = 4, biological replicates). (Q) Representative IGV browser tracks of Tfap4 ChIP-seq data showing binding peaks across the promoter region of Itga11. Primers used for qPCR validation of Tfap4 occupancy at these regions are indicated as Peak1 (P1), Peak2 (P2), and Peak3 (P3). TSS, transcription start site. (R) ChIP-qPCR showing that Tfap4 binds to Itga11 promoter region in NIH 3T3 cells. IgG was used as a negative control (*n* = 3, technical replicates). (S) Schematic representation of the Itga11 promoter region (−2000 to −0 bp). The Tfap4 binding site (−1369 to −1378 bp) was marked. (T) Dual-Luciferase reporter assay showing activation of Itga11 by *Tfap4* overexpression in NIH 3T3 cells (*n* = 3, biological replicates). All experiments were repeated at least three times. Data are presented as mean ± SEM. Groups were compared using two-tailed unpaired *t*-test ((G)-(H) and (L)-(N)), one-way ANOVA with Tukey's multiple comparisons test (T) or two-way ANOVA with Sidak's multiple comparisons test ((P) and (R)). ^∗^*P* ​< ​0.05, ^∗∗^*P* ​< ​0.01, and ^∗∗∗^*P* < 0.001.

Based on our RNA-seq analyses, we discovered that key mechanotransduction pathways, including cell adhesion, PI3K-AKT signaling, cellular response to mechanical stimulus and calcium signaling pathways(Di-Luoffo et al., 2021; Mascharak et al., 2024), were upregulated by *Tfap4* overexpression (Fig. 3(B)–(C)). We therefore sought to explore the role of Tfap4 in regulating mechanotransduction in CFs. Immunoblotting analysis showed that Tfap4 overexpression led to increased levels of phosphorylated and total AKT, along with elevated expression of fibrosis marker Fibronectin and Vimentin (Fig. 4(A)). At the transcription level, genes regulated by PI3K-AKT pathway (e.g., *Fgf1* and *Itga11*)(Wang et al., 2019; Zhang et al., 2021), and those involved in Ca^2+^ regulation (e.g., *Cacna1a* and *Slc8a1*)(Striessnig et al., 2014; Xue et al., 2023) and mechanical response (e.g., *Bmp6* and *Piezo2*)(da Silva Madaleno et al., 2020; Xiao, 2024) were also remarkably increased in Tfap4-CFs (Fig. 4(B)-(D)).

Mechanical stress in CFs is primarily sensed and transduced by focal adhesions (FAs) and associated cytoskeletal proteins, with integrins being among the most extensively studied adhesion receptors for transmitting extracellular mechanical signals to the cytoskeleton. Recently, DNA-based tension probes(Li et al., 2021; Wang et al., 2023) were developed, enabling us to measure real-time mechanical responses in living CFs upon Tfap4 manipulation. In our experiments, CFs were plated onto coverslips coated with a 17-pN DNA-based tension probe to establish an optimal integrin-generated force. We then used reflection interference contrast microscopy (RICM) and total internal reflection fluorescence microscopy (TIRF) to visualize and quantify tension signals (Fig. 4(E)). RICM imaging revealed that control cells spread on the substrate in a rod-like shape, while Tfap4-CFs exhibited a markedly reduced spreading area, suggesting altered cell mechanics (Fig. 4(F)–(G)). Consistently, a pronounced ring-like distribution of strong tension signals was observed in Tfap4-CFs (Fig. 4(H)), indicating an enhanced mechanical response under these conditions.

Next, we stained CFs with F-actin and Paxillin to visualize filamentous actin and focal adhesion respectively. Compared to control cells, which exhibited sparse and elongated FAs with some thicker filaments extending toward the cell center, Tfap4-CFs displayed a markedly different cytoskeletal architecture. In Tfap4-CFs, actin filaments were organized into prominent double-ring structures at the cell periphery, and FAs appeared as dense, stable puncta (Fig. 4(I)). This structure is known to enhance FA stability, promote fibroblast adhesion, and facilitate more effective transmission of mechanical signals(Li et al., 2021). Quantitative analysis further revealed significantly increased F-actin intensity in Tfap4-CFs, with a more centralized distribution, consistent with enhanced cytoskeletal organization and mechanotransduction (Fig. 4(J)). Taken together, Tfap4 overexpression in CFs leads to significant changes in mechanotransduction, likely contributing to fibrosis by sensitizing CFs to mechanical stimuli.

### 2.5 Mechanosensor Itga11 is involved in Tfap4-mediated fibroblasts activation

Further RNA-seq analysis revealed that integrin alpha-11 (Itga11), a reported mechanosensor(Pesce et al., 2023b; Romani et al., 2021b), was increased in pathways related to adhesion, ECM-receptor interaction, PI3K-AKT signaling pathway and regulation of actin cytoskeleton (Fig. 4(K)). This was confirmed by qPCR analysis, which showed that overexpression of Tfap4 enhanced *Itga11* expression by approximately 4-fold, while its knockdown had the opposite effect (Fig. 4(L)–(M)). To investigate whether Itga11 is a downstream factor bridging Tfap4 and fibrosis, we designed shRNAs targeting Itga11 that achieved efficient ablation (Fig. 4(N)). We found that knockdown of *Itga11* reduced expression of fibrotic genes including *Postn*, *Ctgf* and *Fn1* (Fig. 4(N)) and inhibited AKT phosphorylation (Fig. 4(O)), suggesting that Itga11 positively regulates fibrosis.

Next, we introduced sh*Itga11* into Tfap4-CFs and control CFs. We found that inhibition of *Itga11* attenuated collagen-gel contraction, and partially suppressed Tfap4-induced collagen contraction (Fig. 4(P)). To explore whether Tfap4 directly regulates Itga11 expression, we performed a chromatin immunoprecipitation (ChIP) assay with antibody against Tfap4, followed by qPCR to examine the enrichment of bound Itga11 DNA fragments. We used the publicly available Tfap4 ChIP-seq data (GSE80669)(Chou et al., 2016), to design qPCR primers (Fig. 4(Q)). Indeed, we found significant enrichment of Tfap4 at the promoter regions of Itga11 (Fig. 4(R)). Luciferase assays confirmed that Tfap4 directly binds to Itga11 promoter region, approximately at −1369 to −1378 base pairs (bp) upstream from the transcription start site (TSS) (Fig. 4(S) and (T)). Tfap4 failed to activate the luciferase expression driven by a truncated Itga11 promoter lacking Tfap4 binding motif. These findings suggest that mechanosensor Itga11 contributes to fibroblast activation by Tfap4.

### 2.6 Revoking Piezo2-dependent Ca^2+^ response by TFAP4 further enhances fibrosis

**Fig. 5:**
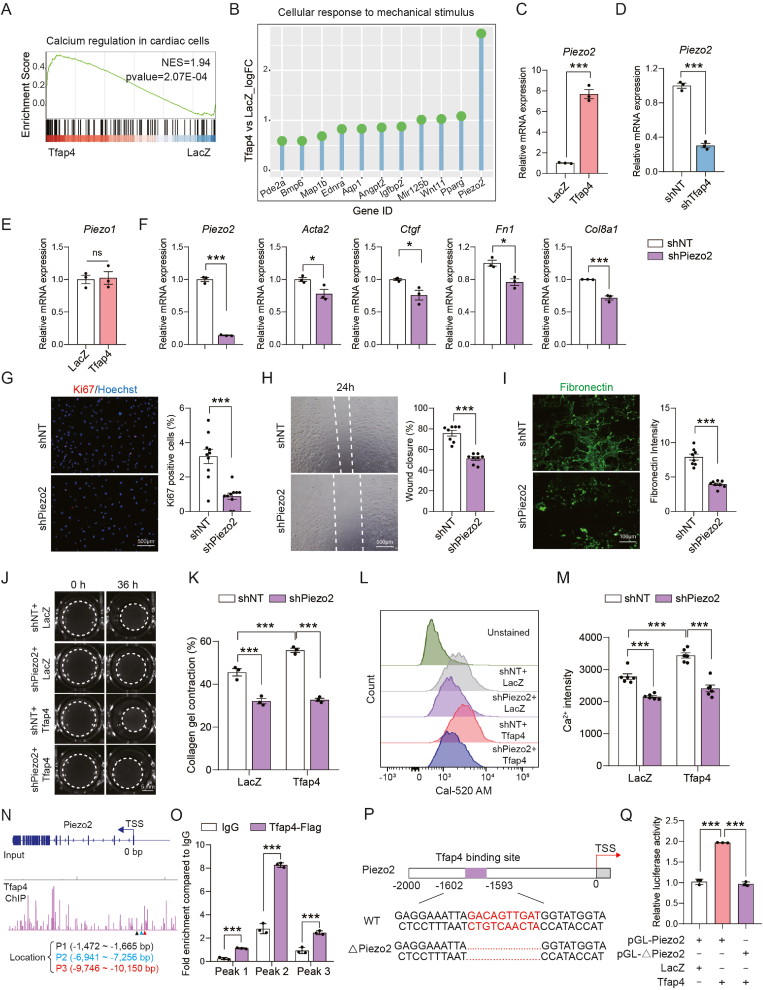
**Piezo2 was identified as a direct downstream target of Tfap4 that regulates fibrotic response in CFs.** (A) GSEA analysis showing the enrichment of the gene set associated with calcium regulation in cardiac cells in Tfap4-CFs compared to LacZ control cells. (B) Lollipop Chart showing DEGs enriched in the cellular response to mechanical stimulus term. (C) RT-qPCR analysis showing *Piezo2* expression in control and Tfap4-CFs (*n* = 3, technical replicates). (D) RT-qPCR analysis showing *Piezo2* expression in CFs treated with shNT or sh*Tfap4* (*n* = 3, technical replicates). (E) RT-qPCR analysis showing *Piezo1* expression in control and Tfap4-CFs (*n* = 3, technical replicates). (F) RT-qPCR analysis showing the expression of *Piezo2* and marker genes for activated fibroblasts in CFs at 5 days post lentiviral shNT or sh*Piezo2* transduction (*n* = 3, technical replicates). (G) Representative ICC images and quantification of Ki67^+^ CFs that were transduced with lentiviruses harboring shNT or sh*Piezo2* (*n* = 10, biological replicates). Scale bar, 500 μm. (H) Representative images (left) and quantification (right) of the wound healing rate 24 h after a scratch in CFs subjected to transduction of shNT or sh*Piezo2* (*n* = 8, technical replicates). Scale bar, 500 μm. (I) Representative ICC images and quantification showing the deposition of fibronectin in CFs treated with shNT or sh*Piezo2* (*n* = 8, technical replicates). Scale bar, 100 μm. ((J)–(K)) Representative bright-field images (J) and quantification (K) showing contraction area of floating collagen gels embedded with control- or Tfap4-CFs treated with shNT or sh*Piezo2* at indicated time points (*n* = 3, biological replicates). Scale bar, 5 mm. ((L)–(M)). Intracellular calcium levels in control- or Tfap4-CFs treated with shNT or sh*Piezo2* were determined using calcium indicator Cal-520 a.m. by flow cytometry (L) and Ca^2+^ average fluorescence intensity was measured by FlowJo (M) (*n* = 6, biological replicates). (N) Representative IGV browser tracks of Tfap4 ChIP-seq data showing binding peaks across the promoter region of Piezo2. Primers used for qPCR validation of Tfap4 occupancy at these regions are indicated as Peak1 (P1), Peak2 (P2), and Peak3 (P3). TSS, transcription start site. (**O**) ChIP-qPCR showing that Tfap4 binds to *Piezo2* promoter region in NIH 3T3 cells. IgG was used as a negative control (*n* = 3, technical replicates). (P) Schematic representation of the Piezo2 promoter region (−2000 to 0 bp). The Tfap4 binding site (−1593 to −1602 bp) was marked. (Q) Dual-Luciferase reporter assay showing activation of Piezo2 by *Tfap4* overexpression in NIH 3T3 cells (*n* = 3, biological replicates). All experiments were repeated at least three times. Data are presented as mean ± SEM. Groups were compared using two-tailed unpaired *t*-test ((C)–(I)), one-way ANOVA with Tukey's multiple comparisons test (Q) or two-way ANOVA with Sidak's multiple comparisons test ((K), (M) and (O)). ^∗^*P* ​< ​0.05, ^∗∗^*P* ​< ​0.01, and ^∗∗∗^*P* < 0.001.

Ca^2+^ signaling in cardiac fibroblasts is crucial for mechano-transduction and fibrosis(Adapala et al., 2013; Blythe et al., 2019). GSEA revealed that *Tfap4* overexpression significantly upregulated pathways related to calcium regulation (Fig. 5(A)). Piezo channels have been reported to transduce mechanical stimuli into intracellular signals, typically increasing intracellular Ca^2+^ levels to regulate fibrosis(Coste et al., 2010). Among these factors, RNA-seq analysis identified *Piezo2* as significantly upregulated in Tfap4-CFs (Fig. 5(B)). This was further validated by RT-qPCR, which confirmed that Tfap4 overexpression increased Piezo2 expression, while its knockdown resulted in a marked reduction (Fig. 5 (C) and (D)). While *Piezo1* has been widely recognized as a key fibroblast activator(Blythe et al., 2019; Niu et al., 2022), our data revealed that TFAP4 overexpression had minimal impact on *Piezo1* expression (Fig. 5(E)).

To investigate whether Piezo2 affects fibroblast activation, we constructed specific shRNAs targeting *Piezo2* achieving 85% knockdown efficiency (Fig. 5(F)). We found that *Piezo2* deficiency significantly inhibited the expression of maker genes in fibroblast activation (Fig. 5F). At the cellular level, *Piezo2* knockdown decreased fibroblast proliferation, migration, and ECM deposition, suggesting its pivotal role in regulating fibrosis (Fig. 5(G)–(I)).

We then knocked down *Piezo2* in Tfap4-CFs and tested their ability to contract. Our results showed that sh*Piezo2* led to reduced contraction and further attenuated the increased contraction induced by Tfap4 (Fig. 5(J)–(K)). Furthermore, using flow cytometry assay to evaluate intracellular Ca^2+^ content in cells stimulated with TGF-β, we found that sh*Piezo2* decreased Ca^2+^ influx, while overexpression of Tfap4 significantly increased it. Interestingly, knockdown *Piezo2* further reduced the Tfap4-induced Ca^2+^ influx (Fig. 5(L)–(M)). These results indicated that Piezo2 was a downstream mediator for Tfap4-induced fibrosis.

Next, we sought to understand if Tfap4 directly regulates Piezo2 expression. Similar to Itga11, we used the same Tfap4 ChIP-seq data to design three pairs of qPCR primers (Fig. 5(N)). We then performed ChIP-qPCR, which revealed that Tfap4 directly bound to these promoter regions (Fig. 5(O)). Consistent with these findings, luciferase assay revealed that Tfap4 directly activated the Piezo2 promoter and increased its transcriptional activity (Fig. 5(P)–(Q)). In comparison, Tfap4 failed to activate the truncated piezo2 promoter that lacks its binding sequence (Fig. 5(Q)). Together, these results suggest that activation of Piezo2 plays a crucial role in Tfap4-induced fibrosis.

### 2.7 Loss of Tfap4 mitigates post-MI cardiac fibrosis and improves cardiac function

**Fig. 6:**
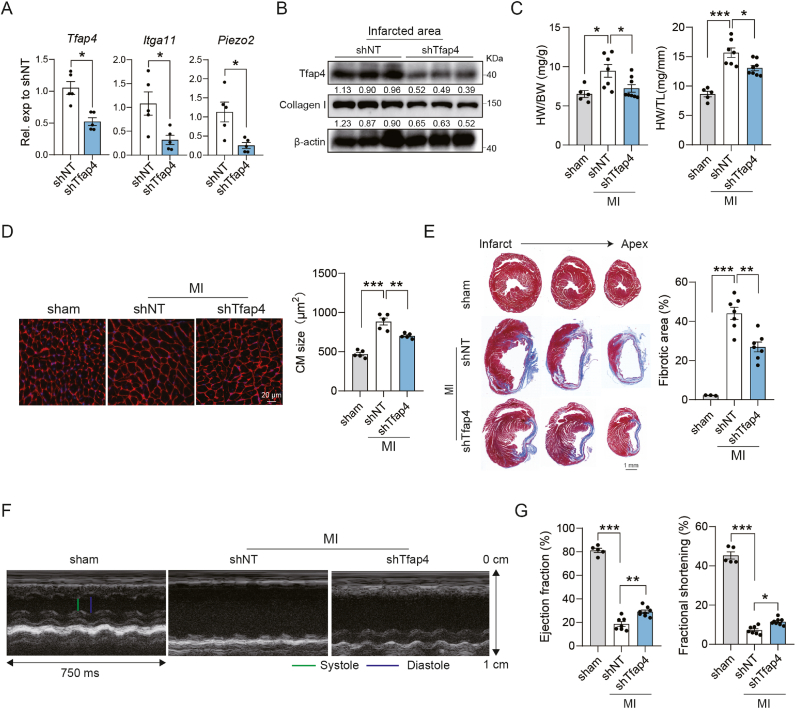
**Tfap4 deletion protects mice againstMI-induced cardiac fibrosis.** (A) RT-qPCR analysis showing *Tfap4*, *Itga11* and *Piezo2* expression in the infarcted area at 4 weeks post-MI (*n* = 5, biological replicates). (B) Western blot and quantification showing Tfap4 and Collagen I in the infarcted area of shNT or sh*Tfap4* group at 4 weeks post-MI (*n* = 3, biological replicates). (C) The ratio of heart weight to body weight (HW/BW) and heart weight to tibia length (HW/TL) of three mice groups (sham, MI-shNT and MI-sh*Tfap4*) (*n* = 5 for sham group; *n* = 7 for shNT and *n* = 8 for sh*Tfap4* group, biological replicates). (D) Representative images and quantitative evaluation of cardiomyocyte (CM) size by wheat germ agglutinin (WGA) staining of three mice groups (sham, MI-shNT and MI-sh*Tfap4*) (*n* = 5, biological replicates). Scale bar, 20 μm. (E) Representative histological heart sections with Masson trichrome staining and quantification showing the areas of fibrosis among indicated groups (*n* = 3 for sham group; *n* = 7 for shNT and *n* = 7 for sh*Tfap4* group, biological replicates). Scale bar, 1 mm. (F) Representative echocardiograms of mice hearts at four weeks post-MI. (G) Quantification of ejection fraction, fractional shortening at 4 weeks after MI (*n* = 5 for sham group; *n* = 7 for MI-shNT group and *n* = 8 for MI-sh*Tfap4* group, biological replicates). All experiments were repeated at least three times. Data are presented as mean ± SEM. Groups were compared using two-tailed unpaired *t*-test (A) or one-way ANOVA with Tukey's multiple comparisons test ((C)-(E) and (G)). ^∗^*P* ​< ​0.05, ^∗∗^*P* ​< ​0.01, and ^∗∗∗^*P* < 0.001.

To determine whether Tfap4 regulates post-MI cardiac remodeling in vivo, we knocked down *Tfap4* in mouse hearts using a lentivirus-mediated gene delivery approach, as previously described(Xie et al., 2023). Knockdown efficiency was confirmed by RT-qPCR and Western blot (Fig. 6(A)–(B)). Consistently, *Tfap4* loss markedly reduced *Itga11* and *Piezo2* expression in MI hearts (Fig. 6(A)). Pathological remodeling following MI was evident by a significant increase in heart weight/body weight and heart weight/tibia length ratios, both of which were reduced by *Tfap4* deficiency at 4 weeks post-MI (Fig. 6(C)). Wheat germ agglutinin staining revealed a reduced cardiomyocyte cross-sectional area in mice with *Tfap4* knockdown (Fig. 6(D)). Histological analyses showed decreased fibrotic area, as assessed by Masson's trichrome staining (Fig. 6(E)), accompanied by reduced collagen I deposition, confirmed by Western blot (Fig. 6(B)).

To evaluate the functional impact of *Tfap4* deficiency, we performed echocardiography (Fig. 6(F)). At 4 weeks post-MI, left ventricular ejection fraction (EF) and fractional shortening (FS) were significantly reduced in MI hearts compared with the sham group. Notably, *Tfap4* deficiency improved both EF and FS compared to controls (Fig. 6(G)). Together, these findings demonstrate that *Tfap4* deletion mitigates adverse post-MI remodeling by attenuating myocardial fibrosis and preserving cardiac function.

### 2.8 TFAP4 regulates pro-fibrotic response of human fibroblasts

**Fig. 7:**
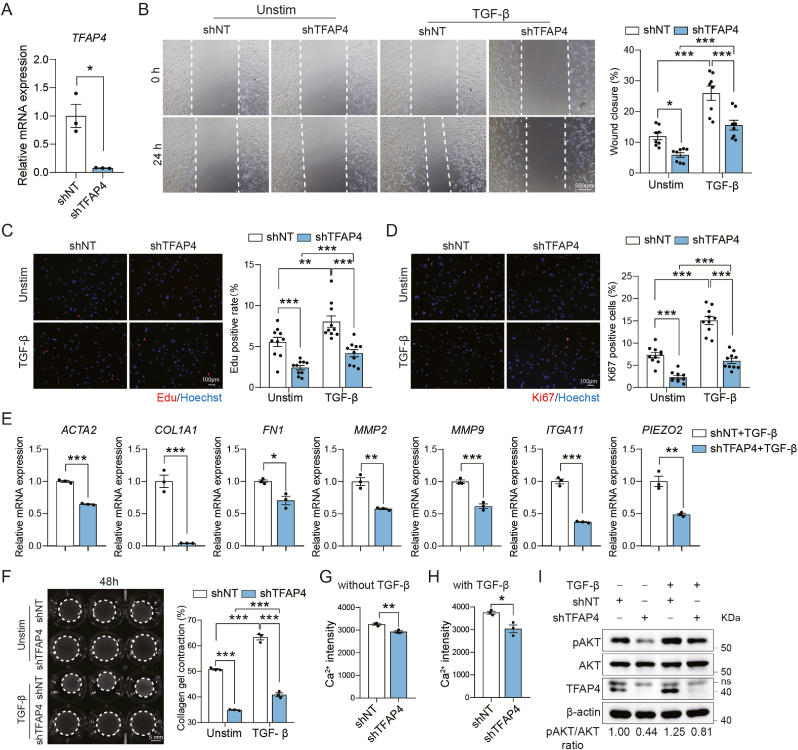
**Tfap4 regulates HCF activation.** (A) RT-qPCR analysis showing the expression of *TFAP4* in HCFs treated with shNT or sh*TFAP4* (*n* = 3, technical replicates). (B) Measurement of cell migration capacity in HCFs treated with shNT or sh*TFAP4* under unstimulated or TGF-β conditions in a wound healing assay. Representative images (left) with quantification of the wound closure percentage (right) 24 h were shown (*n* = 8, technical replicates). Scale bar, 100 μm. (**C** and **D**) Representative ICC images and quantification of Edu^+^ (C) and Ki67^+^ (D) CFs that were transduced with lentiviruses harboring shNT or sh*TFAP4* under unstimulated or TGF-β conditions (*n* = 10, biological replicates). Scale bar, 100 μm. (E) RT-qPCR analysis showing the expression of marker genes for activated fibroblasts in CFs at 5 days post lentiviral shNT or sh*TFAP4* transduction under TGF-β treatment (*n* = 3, technical replicates). (F) Representative bright-field images (left) and quantification (right) showing contraction area of floating collagen gels embedded with shNT or sh*TFAP4* in unstimulated or TGF-β conditions at indicated time points (*n* = 3, biological replicates). Scale bar 5 mm. (G) Intracellular calcium levels in HCFs transduced with shNT or sh*TFAP4* were assessed using the calcium indicator Cal-520 a.m. and analyzed by flow cytometry. Mean fluorescence intensity (MFI) of Ca^2+^ was quantified using FlowJo (*n* = 3 biological replicates). (H) Intracellular calcium levels in HCFs under TGF-β treatment, with either shNT or sh*TFAP4*, were similarly assessed by Cal-520 a.m. staining and flow cytometry. Ca^2+^ MFI was quantified using FlowJo (*n* = 3 biological replicates). (I) Western blot and quantification showing pAKT and AKT expression in HCFs treated with shNT or sh*TFAP4* under unstimulated or TGF-β conditions. All experiments were repeated at least three times. Data are presented as mean ± SEM. Groups were compared using two-tailed unpaired *t*-test or two-way ANOVA with Sidak's multiple comparisons test ((B)-(D) and (F)). ^∗^*P* ​< ​0.05, ^∗∗^*P* ​< ​0.01, and ^∗∗∗^*P* < 0.001.

Having established the role of Tfap4 in murine cardiac fibroblasts, we then investigated its function in HCFs. Sequence alignment showed that TFAP4 was highly conserved among rodents, non-human primates and humans (Fig. S5 A and B). We introduced shRNA targeting *TFAP4* to HCFs, achieving 90% knockdown efficiency as evaluated by RT-qPCR (Fig. 7(A)). Similar to murine CFs, knocking down *TFAP4* contributed to attenuated migration and proliferation of HCFs induced by TGF-β (Fig. 7(B)-(D)). RT-qPCR further showed that *TFAP4* knockdown significantly inhibited the expression of genes involved in HCF activation, including *ACTA2*, *COL1A1*, *FN1*, *MMP2* and *MMP9* (Fig. 7(E)). Importantly, *TFAP4* knockdown also significantly inhibited *ITGA11* and *PIEZO2* expression upon TGF-β treatment (Fig. 7(E)). In a gel contraction assay, *TFAP4* deficiency was associated with a significant decrease in HCF contraction with or without TGF-β treatment (Fig. 7(F)). Additionally, *TFAP4* silencing resulted in reduced fibrosis and decreased intracellular Ca^2+^ levels in HCFs under TGF-β stimulation (Fig. 7(G) and (H)). At the protein level, phosphorylation of AKT was dramatically inhibited by sh*TFAP4*, suggesting a reduced PI3K-AKT pathway activation (Fig. 7(I)). These findings suggest that TFAP4 plays a conserved, pro-fibrotic role in controlling human fibroblast activation.

## 3 Discussion

In this study, we revealed the pivotal role of TFAP4 in regulating cardiac fibrosis through mechanotransduction pathways. By examining both murine and human cardiac fibroblasts, we established that TFAP4 is highly conserved across species and functions as a crucial mediator in fibroblast activation and the fibrotic response. Loss of Tfap4 in vivo ameliorated post-MI cardiac fibrosis and improved cardiac function, highlighting its potential as a therapeutic target for cardiac remodeling and dysfunction.

TFAP4, a member of the bHLH family, has been extensively studied in cancer but remains poorly characterized in fibrosis and tissue remodeling(Mermod et al., 1988; Wong et al., 2021). Our study reveals a previously unrecognized role for TFAP4 in cardiac fibrosis, demonstrating its accumulation in activated cardiac fibroblasts both in vitro and in vivo. TFAP4 overexpression promoted fibroblast proliferation and upregulated pro-fibrotic genes, including *FN1* and *VIM*. Notably, we identified mechanotransduction molecules *Itga11* and *Piezo2* as novel TFAP4 targets, linking TFAP4 to fibrosis through mechanical signaling. Recent studies further support the pro-fibrotic role of TFAP4, showing its activation of NK-1R in renal fibrosis and STING signaling in liver fibrosis(Han et al., 2025; Zhu et al., 2023). In vivo knockdown of *Tfap4* resulted in reduced scar size and improved heart function, further suggesting its critical role in fibrosis. However, we acknowledge that the use of lentivirus-mediated shRNA delivery system may lack cell-type specificity, preventing precise attribution of TFAP4's effects in vivo. Using snRNA-seq analysis of human heart disease samples, we revealed that TFAP4 is expressed in diverse cardiac cell types such as cardiomyocytes and endothelial cells. TFAP4 may also participate in mechanotransduction pathways in those cardiac cell types. Future studies using conditional knockout models will be essential to dissect its cell-type-specific contributions of Tfap4 to fibrosis and cardiac repair.

Mechanotransduction plays a central role in the initiation and propagation of fibrosis(Mascharak et al., 2024). Prolonged excessive mechanical stimulation can result in organ fibrosis. Regulating mechanical force during the transformation of CFs into myofibroblasts may mitigate the adverse effects of fibrosis, reducing myocardial stiffness after MI and thereby preventing heart failure(Yong et al., 2015; Younesi et al., 2024). By performing unbiased transcriptomic analyses, we identified that *Tfap4* overexpression activates key mechanotransduction-related pathways such as PI3K-AKT signaling, integrin/FAs and calcium signaling. These findings were further validated by an increase in phosphorylated AKT levels and downstream target gene expression. Importantly, leveraging our recently developed DNA-based tension probe, we discovered that TFAP4 sensitizes CF to mechanical stimuli, thereby promoting fibrosis. These results suggest that TFAP4's modulation of mechanical signaling could serve as a potential mechanism to exacerbate fibrotic remodeling in the myocardium.

Fibroblasts can sense mechanical forces through mechanosensitive receptors. Crucial mechanosensors, including integrins(Niu et al., 2022), mechanosensitive ion channels(Adapala et al., 2013; Du et al., 2010), and G protein-coupled receptors (GPCRs)(Zhang et al., 2022) are reported to promote fibrosis in response to mechanical signals. In our study, we identified Itga11 and Piezo2 as direct TFAP4 targets that mediate fibroblast activation. The α11 integrin has been reported to be expressed by cardiac fibroblasts and binds preferentially to type I collagen fibers(Talior-Volodarsky et al., 2015). Early studies have demonstrated that *Itga11* deletion attenuates cardiac fibrosis and improves cardiac function in diabetic mice(Civitarese et al., 2016; Romaine et al., 2018). Especially, *Itga11* knockdown impairs the contractility of collagen I matrices in liver fibroblasts, which aligns with our discoveries. We found that knockdown *Itga11* partially mitigated TFAP4-induced fibrosis activation, as shown by reduced pro-fibrotic gene expression, lower phosphorylated AKT level and impaired contractility. Piezo2 is a mechanosensitive Ca^2+^ channel that plays a significant role in cardiovascular health and disease(Beech & Kalli, 2019). We found that TFAP4 directly activated *Piezo2*, aligning with recent findings that *Piezo2* expression increases in activated fibroblasts through YTHDF1-mediated m^6^A modification(Ding et al., 2024). Fibroblast-specific *Piezo2* deficiency reduced fibroblast activation, autophagy, and cardiac fibrosis(Ding et al., 2024). Our study further identified Tfap4 as a direct transcriptional activator for Piezo2, highlighting its role in modulating mechanosensitive pathways in fibrosis.

Yes-associated protein (YAP) and transcriptional coactivator with PDZ-binding motif (TAZ) are central components of the Hippo signaling pathway and serve as well-characterized downstream effectors in mechanotransduction. In response to mechanical cues such as increased ECM stiffness or TGF-β signaling, YAP/TAZ translocate into the nucleus where they partner with transcription factors such as TEAD to drive the expression of profibrotic and proinflammatory genes(Cho et al., 2025; Garoffolo et al., 2022; Mia et al., 2022). While both TFAP4 and YAP/TAZ are implicated in fibrosis, they function at distinct regulatory levels. TFAP4 appears to act upstream in the signaling cascade, functioning as a transcriptional initiator of mechanosensor expression and mechanosensory signaling. In contrast, YAP/TAZ serve as downstream effectors that amplify and execute fibrotic transcriptional programs in response to those mechanical signals. Thus, TFAP4 may represent an early regulatory "switch" that enables cellular perception of the mechanical environment, whereas YAP/TAZ execute context-dependent fibrotic responses.

Together, we demonstrated that Tfap4 promotes cardiac fibrosis through the direct activation of mechanosensors, including Itga11 and Piezo2, which in turn activate key mechanotransduction pathways. Our findings suggest that targeting TFAP4 could provide a novel therapeutic approach to mitigate fibrosis and improve cardiac functions in patients with myocardial infarction.

## 4 Materials and methods

### 4.1 Animals

C57BL/6J mice at 6–8 weeks old were purchased from Beijing Vitalstar Biotechnology Co., Ltd. (Beijing, China).

### 4.2 Plasmid construction

Tfap4 was amplified from the cDNA of adult mouse spleen by PCR and cloned into the pLenti vector (Addgene #39481) and pHAGE-puro vector with 3xFlag tag (kindly gifted from Dr. Jing Zhang Lab in Wuhan University). Cloning primers are listed in Supplemental Table 1. To silence gene expression, shRNA oligos from Sigma were annealed to pLKO.1 vector as previously described(Wang et al., 2020). Sequence information of shRNA oligos used in this study is listed in Supplemental Table 2. For the luciferase assay, the promoter sequences of Piezo2 and Itga11 were amplified from the genomic DNA from mouse heart. The amplified PCR products were then inserted into the pGL3-enhancer vector (Addgene #212938) to produce the pGL3-Piezo2 and pGL3-Itga11 plasmid respectively. The truncated constructs, pGL3-△Itga11 and pGL3-△Piezo2, were made by depleting Tfap4 binding sequences within the promoter region. Cloning primers are listed in Supplemental Table 1.

### 4.3 Isolation of primary murine cardiac fibroblasts (CFs) and viral infection

Thy1.2^+^ CFs were isolated by magnetic-activated cell sorting (MACS) using standard protocol described previously(Wang et al., 2015). Isolated CFs were seeded onto 0.1% gelatin-coated plates in fibroblast medium containing IMDM (Hyclone, SH30228.01) supplemented with 20% FBS and 1% penicillin/streptomycin at a density of 2e4 to 3e4 cells per cm^2^. Twenty-four hours after seeding, lentivirus (4 μL/well of 24 well plate) was added to the cells. Twenty-four hours after viral infection, CFs were selected with culture medium containing puromycin (1 μg/mL) for three days and were collected on day 5 for downstream analysis.

### 4.4 Maintenance of human CFs (HCFs)

The human primary cardiac fibroblast (HCF) was purchased from the BeNa Culture Collection (BNCC, BNCC354381, Beijing China). HCFs were seeded at density of 1e4 cells per cm^2^ at day 0. On day 1, lentivirus (4 μL/well) was added to the cells. On day 2, CFs were maintained in medium containing puromycin (1 μg/mL) for three days to enrich infected cells. Cells were collected on day 6 for downstream analysis.

### 4.5 Mechanical force visualization assay

Mechanical force visualization was performed as previously described(Li et al., 2021). Circular coverslips were rinsed and sonicated three times in nanopure water and then sonicated in acetone for 20 min, followed by drying in an oven. The dried coverslips were activated in oxygen plasma for 10 min to obtain a hydroxylated surface, then incubated in ethanol with 1% v/v (3-Aminopropyl) triethoxysilane for 1 h for amine modification, rinsed with ethanol and dried under a stream of N_2_. The amine-modified coverslips were passivated by covering with 200 μL of 0.1 M fresh sodium bicarbonate solution containing 5% w/v mPEG-NHS (*M*_w_ = 2000) and 0.5% w/v lipoic acid-PEG-NHS (*M*_w_ = 3400). After incubation at 4 °C overnight, the coverslips were washed with nanopure water and incubated with 16 nM 5 nm AuNPs at room temperature for 30 min. Then, coverslips were rinsed three times with nanopure water to remove non-specifically bound AuNPs. Finally, 200 nM DNA tension probe in PBS (10 mM sodium phosphate, 1 M NaCl, pH 7.2) was added onto the surface and incubated at room temperature for 1 h, and then the non-specifically bound probes were removed by rinsing with PBS solution. The treated coverslips were assembled into cell imaging chambers for cell experiments. All images were acquired using a Nikon Eclipse Ti2 inverted microscope with Nikon LU-N4 laser units equipped with four lasers (405 nm, 488 nm, 561 nm and 640 nm) (15 mW), an autofocus system, a × 100 oil objective (Nikon, 1.49 NA) and an ANDOR EMCCD (1024 × 1024 pixels) camera with Nikon NIS-Elements software. Image data were processed with Image J and the MATLAB.

### 4.6 Chromatin Immunoprecipitation (ChIP) followed with qPCR (ChIP-qPCR)

ChIP was performed as previously described with minor optimizations(Liu et al., 2016). Briefly, 5–10 million NIH 3T3 cells were cross-linked using 1% paraformaldehyde/DPBS at room temperature for 8 min and were sheared using Bioruptor (30 s on/45 s off × 23 cycles). The sheared chromatin DNA–protein complex was then precipitated with anti-Flag antibody (Sigma, F3165) and normal mouse IgG (Santa Cruz Biotechnology, B0619) at 4 °C overnight. The immunoprecipitated products were washed sequentially and eluted in elution buffer (1% SDS, 0.1 M NaHCO_3_) at 68 °C, with agitation at 1400 rpm for 40 min. Decrosslinking was performed by incubation at 42 °C for 2 h and then at 67 °C for 6 h. The DNA was further purified by QIAquick PCR Purification Kit (Qiagen, 28104) and then used for qPCR. Primers were designed with Primer-BLAST and their sequences were listed in Table S4.

### 4.7 Dual-luciferase reporter assay

NIH3T3 cells were co-transfected with 1 μg of empty vector or reporter plasmid harboring target promoters and 0.1 μg of Renilla luciferase vector. At 48 h after transfection, the luciferase activity was measured with a Dual-Glo Luciferase Assay System (Spectramax i3x) and an illuminometer. Renilla luciferase intensity was used as a control to normalize the firefly luciferase intensity.

### 4.8 Bulk RNA sequencing and data processing

After 3 days post infection, cells were collected in TRIzol (Takara) for RNA extraction. RNA quality was determined by examining A260/A280 with NanodropTM OneCspectrophotometer (Thermo Fisher Scientific Inc). RNA Integrity was confirmed by 1.5% agarose gel electrophoresis. Qualified RNAs were finally quantified by Qubit3.0 with QubitTM RNA Broad Range Assay kit (Life Technologies, Q10210). cDNA library was prepared using Ribo-off rRNA depletion Kit (Ribobio, China) and KCTM Stranded mRNA Library Prep Kit for Illumina® (Catalog NO. DR08402, Wuhan Seqhealth Co., Ltd. China) following the manufacturer's instruction. The library products corresponding to 200–500 bps were enriched. Paired-end sequencing was conducted on an Illumina NovaSeq 6000 with a read length of 150 base pairs (bp). Raw sequencing data was initially filtered by Trimmomatic (version 0.36), with the discarding of low-quality reads and the trimming of reads contaminated with adaptor sequences. Subsequently, the clean reads were mapped to the reference genome of mouse (mm10) using STRA software (version 2.5.3a) with default parameters. The counting of reads mapped to the exon regions of each gene was carried out by featureCounts (Subread-1.5.1; Bioconductor) and then RPKMs were calculated. Resulting BAM files were sorted and indexed using SAMtools. Differential expression analysis was performed using DESeq2 (version 1.32.0)(Love et al., 2014), with the criteria for significantly differentially expressed genes (DEGs) setting at fold change ≥1.5 and adjusted *P*value < 0.05. Gene ontology (GO) analysis and Kyoto encyclopedia of Genes and Genomes (KEGG) enrichment analysis for differentially expressed genes were both implemented by DAVID with a *P* value cutoff of 0.05 to judge statistically significant enrichment. Gene Set Enrichment Analysis (GSEA) was performed on R software (version 4.2.2) with a *P* value cutoff of 0.05 to judge statistically significant enrichment.

### 4.9 Transcription factor binding analysis

The prediction of Tfap4 binding sites within promoter regions of Itga11 and Piezo2 was calculated using FIMO (v.4.11.2) software(Grant et al., 2011). The motif file (JASPAR, 2022)(Castro-Mondragon et al., 2022) and promoter sequence of Tfap4 were used as input for the FlMO. A *P* value < 0.001 was selected as the cut-off value for reliable predictions.

### 4.10 Murine model of myocardial infarction (MI)

MI and *in vivo* delivery of lentivirus were performed in 68-week-old male C57BL/6 mice as previously reported(Xie et al., 2023). Mice were anesthetized by intraperitoneal injection of 100 mg/kg body weight (BW) ketamine and 10 mg/kg BW xylazine for one time and were ventilated with an ALC-V8S ventilator. After exposing the heart, MI was performed by permanent ligation of the left anterior descending artery (LAD) with a 7-0 prolene suture. Sham-operated animals served as surgical controls and were subjected to the same procedures as the experimental animals with the exception that the LAD was not ligated. Lentiviruses expressing sh*Tfap4* and shNT were subjected to ultracentrifugation and resuspended in PBS with polybrene (8 μg/mL). Each mouse received 1 × 10^7^ TU lentivirus containing sh*Tfap4* or shNT. Immediately after the ligation, virus-containing solution was injected into the boundary between the infarct zone and border zone at two sites with 31-gauge insulin needles. Mice were allowed to recover on the heating pad after closing the chest. Cardiac function was measured with transthoracic echocardiography using a Vinno 5 imaging system (Vinno, China) 4 weeks after surgery. At the end of the study, all animals were euthanized using CO_2_ asphyxiation, followed by cervical dislocation. MI surgeries, echocardiography and the following analyses were performed in a double-blinded manner. A total of 25 mice were used in this study, with 5 assigned to the sham group, 10 assigned to the MI ​+ ​shNT group and 10 to the MI ​+ ​sh*Tfap4* group. During the post-MI period, 3 and 2 mice died in shNT and sh*Tfap4* group respectively. Most deaths occurred within the first 3 days after MI and were attributed to cardiac rupture, as confirmed by post-mortem examination. Importantly, there was no significant difference in early mortality rates between the groups.

### 4.11 Other assays

Cell culture, viral production, collagen gel contraction assay and other assays are provided in the Supplementary materials.

### 4.12 Statistics analysis

Data were analyzed using Prism 8 (GraphPad Software, La Jolla, CA). All results were expressed as mean ± SEM. The unpaired *t*-test was used to determine the significance of differences between the two groups. One-way or two-way analysis of variance (ANOVA) was used to determine the significance of experiments containing three or more conditions. A value of *P* ​< ​0.05 was considered statistically significant (∗), a *P* value of <0.01 was considered statistically significant (∗∗), a *P* value of <0.001 was considered statistically significant (∗∗∗).

## CRediT authorship contribution statement

**Jie Liu:** Writing – review & editing, Writing – original draft, Visualization, Validation, Methodology, Investigation, Formal analysis, Data curation. **Jingjing Feng:** Writing – original draft, Validation, Investigation, Formal analysis, Data curation. **Jingxuan Zhao:** Writing – review & editing, Validation, Investigation, Formal analysis, Data curation. **Xiangjie Kong:** Writing – review & editing, Software, Resources, Methodology, Data curation. **Zhangyi Yu:** Writing – review & editing, Writing – original draft, Visualization, Validation, Methodology, Formal analysis, Data curation. **Yuanru Huang:** Writing – review & editing, Software, Methodology, Data curation. **Zechun He:** Writing – review & editing, Writing – original draft, Validation, Formal analysis, Data curation. **Mengxin Liu:** Writing – review & editing, Writing – original draft, Validation, Formal analysis, Data curation. **Zheng Liu:** Writing – review & editing, Supervision, Software, Resources. **Zhibing Lu:** Writing – review & editing, Software, Resources, Methodology. **Li Wang:** Writing – review & editing, Writing – original draft, Visualization, Supervision, Project administration, Methodology, Investigation, Funding acquisition, Data curation, Conceptualization.

## Ethics statement

All mice were maintained in specific pathogen-free (SPF) facilities at the Medical Research Institute of Wuhan University. The experimental protocols followed the International Guiding Principles for Biomedical Involving Animals. The protocols for animal experiments were approved by the Institutional Animal Care and Use Committee of the Medical Research Institute of Wuhan University (approval number: MRI2020-LAC68).

## Data availability

All data generated or analyzed during this study are included in the main text or the Supplementary Material. Bulk RNA-seq is available from the NCBI Gene Expression Omnibus (GEO) under accession number GSE261406.

## Declaration of competing interest

The authors declare the following financial interests/personal relationships which may be considered as potential competing interests:Li Wang reports financial support was provided by 10.13039/501100012166National Key Research and Development Program of China. Li Wang reports financial support was provided by 10.13039/501100001809National Natural Science Foundation of China. Li Wang reports financial support was provided by 10.13039/501100003819Natural Science Foundation of Hubei Province. Li Wang reports financial support was provided by Fundamental Research Funds for the Central Universities in China. If there are other authors, they declare that they have no known competing financial interests or personal relationships that could have appeared to influence the work reported in this paper.
