# Supplementary material for: TFAP4 exacerbates pathological cardiac fibrosis by modulating mechanotransduction
Source: Cell Insight. 2025 Jun 2;4(4):100256. doi: 10.1016/j.cellin.2025.100256 (PMC12226091; doi:10.1016/j.cellin.2025.100256)
Supplement: Multimedia component 1 [file mmc1.docx]

**Supplementary material**

**Supplemental Figures and Legends**

Figure S1. Functional screening for transcription factors potentially affecting fibrosis.

Figure S2. The expression level of TFAP4 in the donor and DCM group.

Figure S3. Tfap4 is essential for fibroblast to myofibroblast transformation.

Figure S4. Gene enrichment analysis of downregulated DEGs in CFs with *TFAP4* overexpression.

Figure S5. Tfap4 is a highly conserved transcription factor.

**Supplemental Methods**

Cell culture and viral production

Culture and TGF-β stimulation of CFs

Cell cycle analysis by propidium iodide (PI) staining

Measurement of intracellular Ca^2+^ level

Collagen gel contraction assay

RNA extraction and RT-qPCR

Wound healing assay

Immunofluorescent staining (ICC)

EdU incorporation assay

Western Blots

Histology analysis

Single‑cell transcriptomic datasets analysis

**Supplemental Tables**

Table S1 List of molecular cloning primers

Table S2 List of shRNA oligos

Table S3 List of qPCR primers

Table S4 List of ChIP-qPCR primers

Table S5 List of antibodies

**
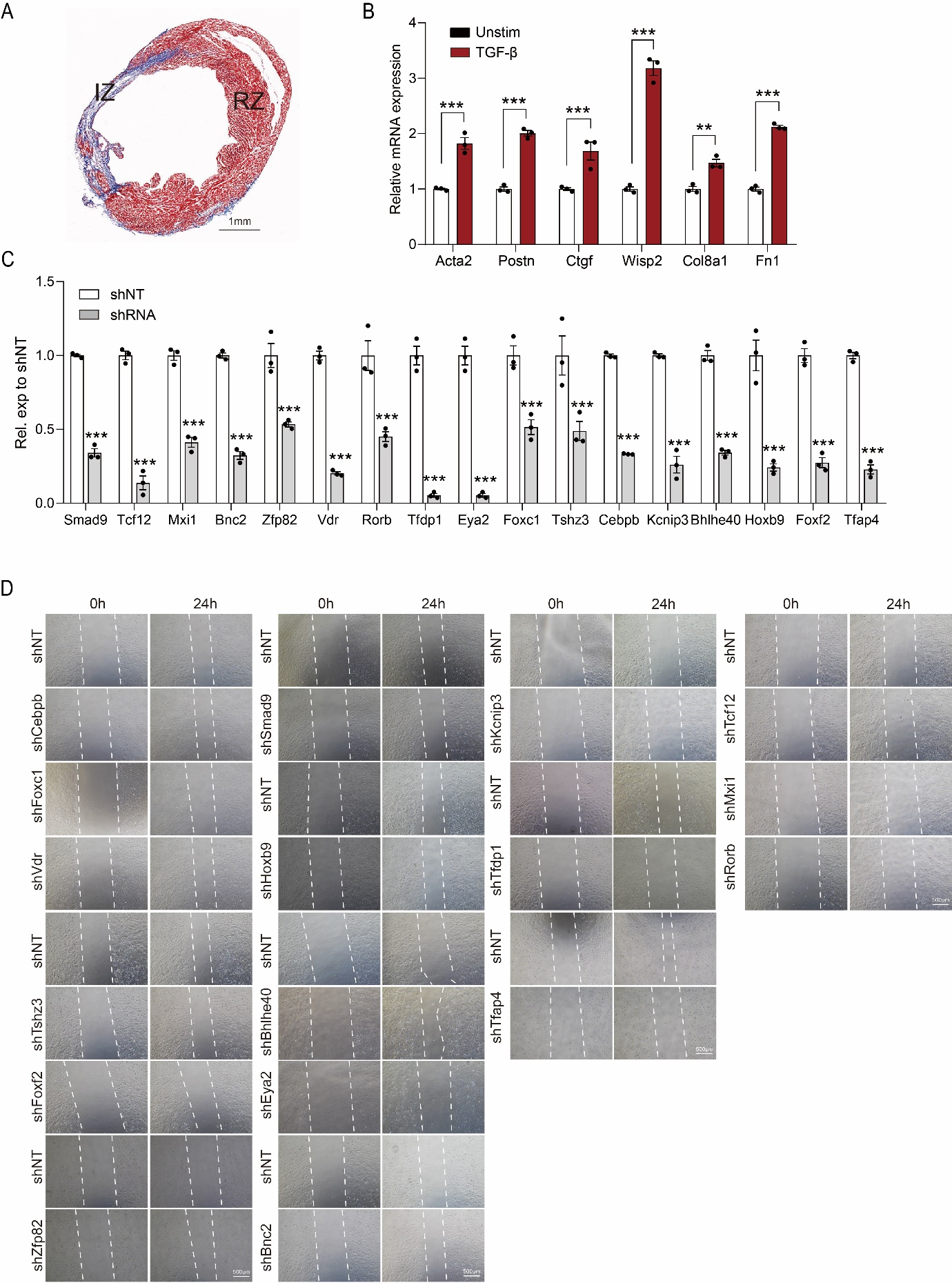
**

**Fig. S1. Functional screening for transcription factors potentially affecting fibrosis.**

(**A**) Representative histological heart sections with Masson trichrome staining 4 weeks post-MI. Remote zone (RZ) and infarct zone (IZ) in infarcted hearts were displayed. Scale bar, 1 mm.

(**B**) RT-qPCR analysis showing the expression of marker genes for activated fibroblasts in CFs under treatment with or without TGF-β (n=3, technical replicates).

(**C**) Knockdown efficiency of shRNAs was determined by RT-qPCR in CFs at 5 days post lentiviral shRNAs targeting indicated transcription factors. Cells transduced with lentiviral shNT were used as control (n=3, technical replicates).

(**D**) Representative bright-field images of the wound healing rate 24 h after a scratch in CFs subjected to transduction of lentiviral shRNAs targeting indicated transcription factors. Scale bar, 500 μm.

All experiments were repeated at least three times. Data are presented as mean ± SEM. Groups were compared using two-way ANOVA with Sidak’s multiple comparisons test. ***P* < 0.01, and ****P* < 0.001.


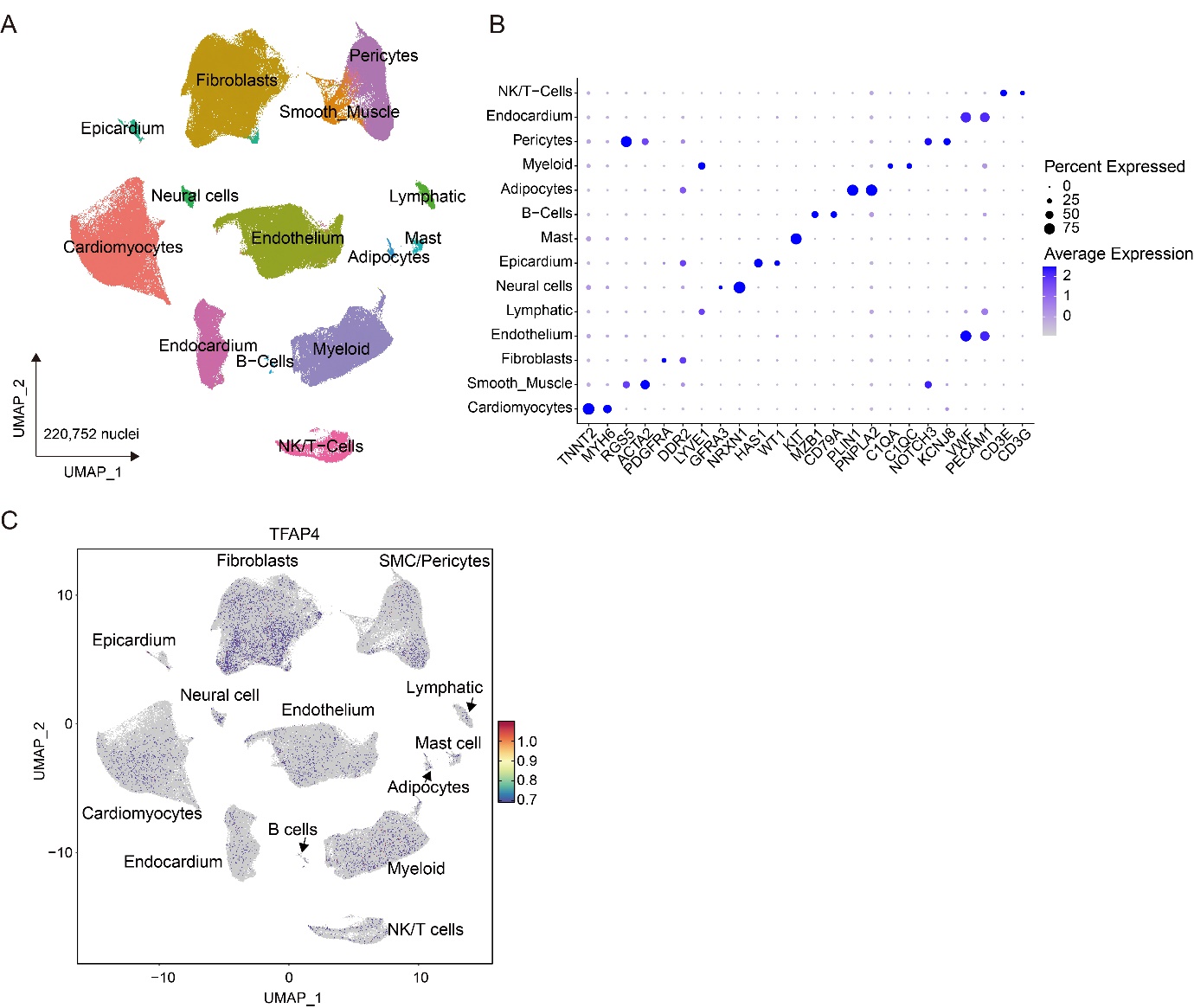


**Fig. S2. The expression level of TFAP4 in the donor and DCM group.**

(**A**) Unsupervised clustering of 220,752 nuclei isolated from healthy donors and DCM hearts identified 14 distinct cell types, visualized in a Uniform Manifold Approximation and Projection (UMAP) plot.

(**B**) Dot plot showing representative marker genes for each identified cell type in the integrated dataset.

(**C**) UMAP visualization of cardiac cells from donor and DCM groups, colored by TFAP4 expression levels.

**
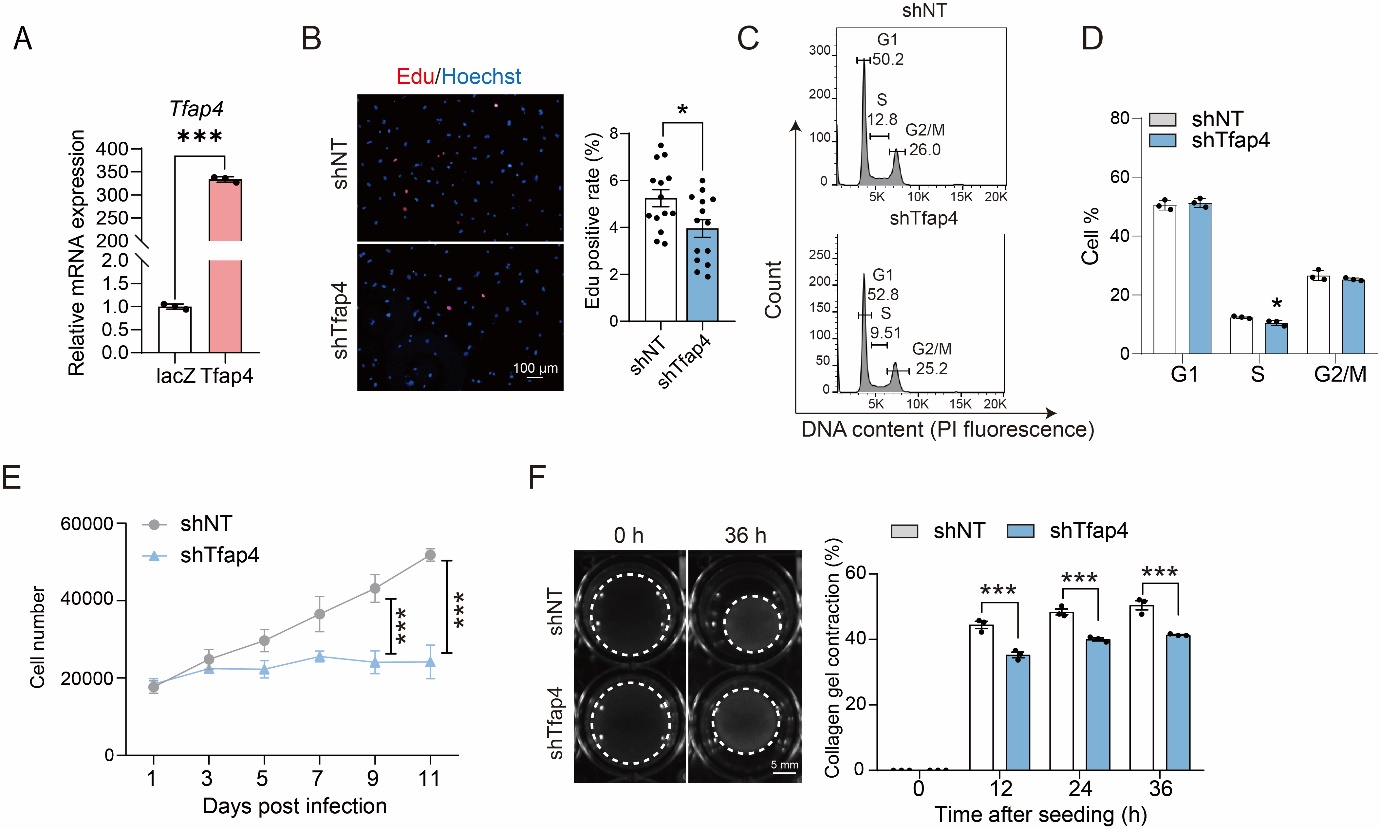
**

**Fig. S3. Tfap4 is essential for fibroblast to myofibroblast transformation.**

(**A**) RT-qPCR analysis showing the overexpression of *Tfap4* in CFs at day 5 post lentiviral transduction. Cells transduced with lentiviral LacZ were used as control (n=3, technical replicates).

(**B**) Representative ICC images and quantification of Edu^+^ CFs that were transduced with lentiviruses harboring control shNT or sh*Tfap4* (n=14, biological replicates). Scale bar, 100 μm.

(**C** and **D**) Representative flow cytometry plots (**C**) and quantification (**D**) of propidium iodide (PI) positive cells distributed in G0/G1, S or G2/M phases. CFs were transduced with lentiviral shNT or sh*Tfap4* and subjected to PI staining at 72 hours post-transduction (n=3, technical replicates).

(**E**) Quantitation of absolute numbers of CFs treated with shNT or sh*Tfap4* from day 1 to day 11 post-viral infection (n=3, technical replicates).

(**F**) Representative bright-field images (left) and quantification (right) showing the contraction area of floating collagen gels embedded with shNT or sh*Tfap4* at indicated time points (n=3, biological replicates). Scale bar, 5 mm.

All experiments were repeated at least three times. Data are presented as mean ± SEM. Groups were compared using two-tailed unpaired *t* test (**A** and **B**) or two-way ANOVA with Sidak’s multiple comparisons test (**D**-**F**). **P* < 0.05, ***P* < 0.01, and ****P* < 0.001.


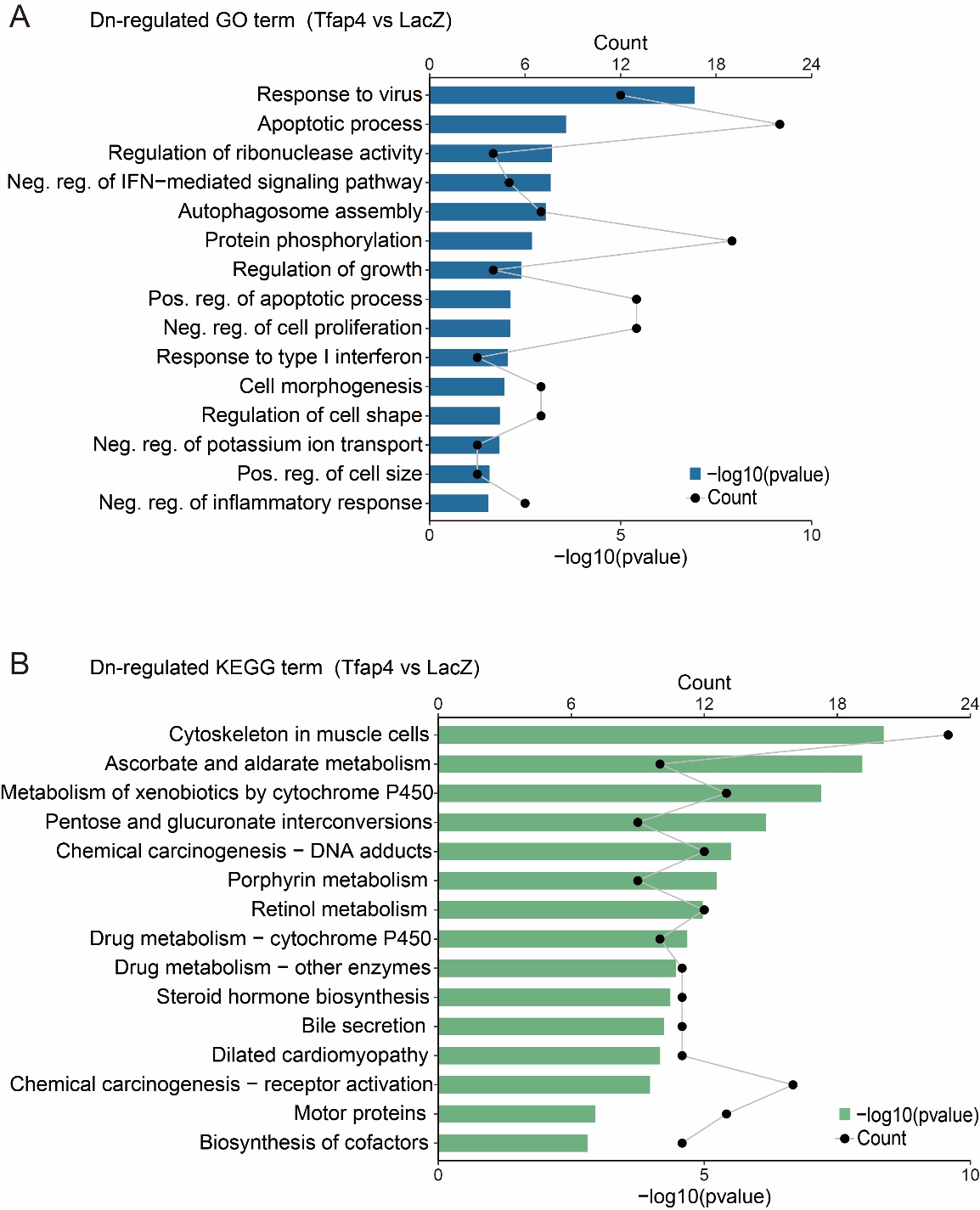


**Fig. S4. Gene enrichment analysis of downregulated DEGs in CFs with *TFAP4* overexpression.**

(**A**) Gene ontology (GO) analysis showing the enriched biological processes associated with DEGs downregulated in Tfap4-CFs compared to LacZ control cells. Neg., Negative; Pos., Positive, reg., regulation.

(**B**) KEGG enrichment analysis showing downregulated signaling pathways in Tfap4-CFs.


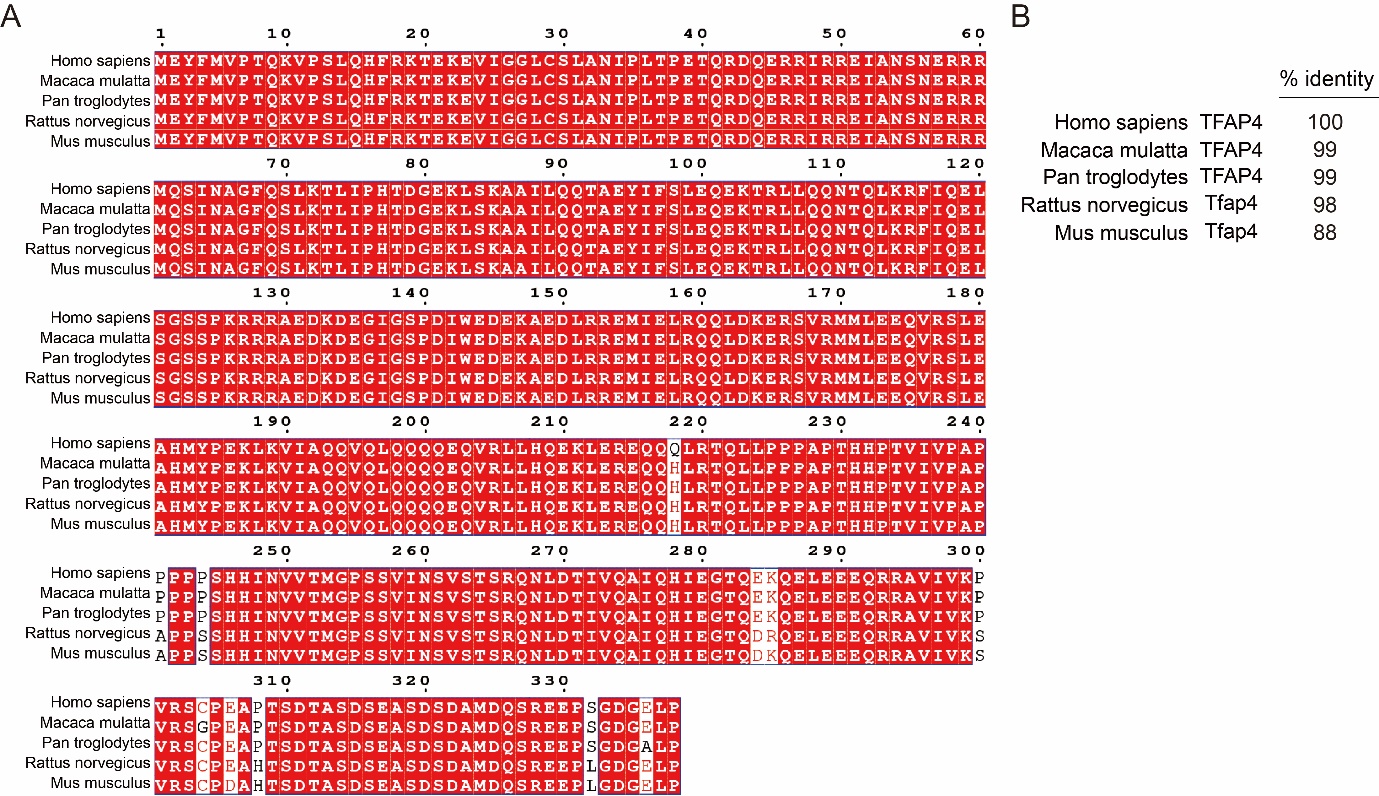


**Fig. S5. Tfap4 is a highly conserved transcription factor*.***

(**A**) Conservation analysis of the TFAP4 protein sequences.

(**B**) Identity of the total sequence between the human TFAP4 protein and TFAP4 proteins from other species.

**Supplemental Methods**

*Cell culture and viral production.*

Human embryonic kidney (HEK) 293T cells (purchased from ATCC) were cultured in Dulbecco’s modified Minimal Essential Medium (DMEM) (Gibco, 6123110) supplemented with 10% fetal bovine serum (FBS) (Newzerum, FBS-AU500) and 1% penicillin/streptomycin (Hyclone, SV30010) as previously described(Wang et al., 2022). One day before transfection, cells were seeded at a density of 4-5 million cells per 10 cm dish. For lentivirus production, 10 µg viral constructs, 7 µg psPAX2 and 3 µg pMD2.G were co-transfected to 293T cells with Polyethylenimine (PEI) (Polysciences, 24765) according to manufacturer’s instructions. Virus-containing culture medium was harvested at 48 and 72 hours after transfection, filtered through 45-μm pore size filters (Millipore) and incubated with PEG6000 solution (8% final concentration) overnight at 4°C. The next day, the mixture was spun down at 3,800 rpm for 30 min at 4℃ and the pelleted virus was resuspended with 100 µL DMEM medium per 10 cm dish. Lentivirus can be used either freshly or frozen at -80 ℃ until further use.

NIH 3T3 cells were purchased from Cell Bank/Stem Cell Bank, Chinese Academy of Sciences and cultured in DMEM supplemented with 10% new-born calf serum (TIANHANG, 22012-0612) and 1% penicillin/streptomycin. Cells were split every 2-3 days.

*Culture and TGF-β stimulation of CFs.*

CFs were seeded at density of 2.5e4 cells per cm^2^ at day 0. On the next day, cells were pretreated with serum-free media overnight and then incubated with TGF-β1 (Peprotech, 100-21C) at a concentration of 10 ng/mL for another 24 hrs as reported17. Cells were then collected for the following analysis.

*Cell cycle analysis by propidium iodide (PI) staining.*

CFs were trypsinized and washed in DPBS and collected by centrifugation at 1,000 rpm for 5 minutes. Cells were then fixed in 70% Ethanol overnight at -20°C. After washed with DPBS, the cells were incubated with FxCycle™ PI/RNase, PI staining solution (ThermoFisher, F10797) at room temperature for 15 min in the dark. Then, cells were resuspended in DPBS and analyzed with flow cytometry (BD FACSCelesta).

*Measurement of intracellular Ca^2+^ level.*

CFs were collected and washed with Hank’s Balanced Salt Solution (HBSS) (Gibco, C14175500BT) and incubated in 5 µM Cal-520 acetomethoxyl methyl ester (AM) (AAT Bioquest, 21130) for 15 min at 37℃ in dark as manufacture suggested. Subsequently, cells were washed with HBSS once, resuspended and incubated in HBSS for 20 min at 37℃ to ensure that Cal-520 AM was completely converted into Cal-520. Finally, the fluorescence intensity of intracellular Cal-520 was assessed by flow cytometry (BD FACSCelesta) and analyzed by FlowJo (v10.8.1).

*Collagen gel contraction assay.*

CFs were collected and mixed with type I collagen solution (Advanced BioMatrix, 5074) at a ratio of 7:3 to create a final fibroblast-collagen gels solution of 4e5 cells/mL. The solution was loaded into 24-well plate and incubated at 37℃ for 1.5 hours to allow collagen polymerization. Gels were then covered with 500 μL fibroblast medium. Images covering the whole well were captured every 12 hours. Gel area for each well was determined using ImageJ software and collagen gel contraction rate was calculated as the percentage of contraction by using the formula: [(initial area - contracted area) / initial area] × 100%.

*RNA extraction and RT-qPCR.*

For RNA extraction, total RNA was isolated using RNAiso Plus (Takara, 9109) and reverse transcription was performed using HiScript II Q RT SuperMix (Vazyme, R222-01). RT-qPCR was performed to quantitatively detect mRNA levels using SYBR Green Master Mix (Yeasen, 11201ES08) on a CFX Connect Real-Time PCR Detection Systems (Bio-rad). The results were normalized to those of GAPDH. The mRNA levels were calculated using the 2–ΔΔCt method. The qPCR primers used in this study are listed in Supplemental Table 3.

*Wound healing assay.*

Wound healing assay was performed as described(Wang et al., 2022). After genetic manipulations, CFs were reseeded into 24-well plate at the density of 4e4 cells per cm^2^. When the density reached 100%, the monolayers of CFs were wounded with a sterile 1-mL plastic pipette tip and then washed three times with DPBS to remove redundant cell debris. The remaining cells were incubated with medium for another 24 h. Images of the scratches were captured on 0 h and 24 h under a light microscope (Nikon). Eight different fields from each sample were considered for quantitative estimation of the distance between the borderlines. The migration rate was calculated as the wound closure percentage using the formula: [(initial wound area - wound area at later time point) /initial wound area] × 100%. The analysis was performed using ImageJ software.

*Immunofluorescent staining (ICC).*

ICC was performed as previously described(Wang et al., 2022). In general, CFs were fixed in 4% paraformaldehyde (PFA) for 15 min at room temperature, permeabilized with 0.1% Triton X-100, and blocked with 5% BSA. Treated cells were then incubated with Vimentin (Progen, GP53, 1:200), Ki67 (Abcam, ab16667, 1:250), F-Actin/ phalloidin-TRITC (Solarbio, CA1610, 1:500), and Paxillin (Abcam, ab32084, 1:500) in 1% BSA overnight. After washing the cells with DPBS three times, cells were incubated with secondary antibodies including Alexa488 (Jackson ImmunoResearch Inc.) or Cy3 (Jackson ImmunoResearch Inc.) for 1 h at room temperature in the dark. After washing, the nuclei were stained with Hoechst 33342 (Biosharp, BL803A). For ECM staining of fibronectin (Proteintech, 15613-1-AP, 1:200), cells were treated as previously described(Wang et al., 2022). The pictures were taken on Nikon and intensity of fluorescence was measured by ImageJ. The antibodies used in this assay were listed in Supplemental Table 5.

*EdU incorporation assay.*

EdU staining was performed using the Cell-Light EdU Apollo567 In Vitro Kit (RiboBio, C10310-1) in accordance with the manufacturer’s instructions. CFs were seeded at the density of 1.5e4 cells per cm^2^ and incubated with 50 µM EdU for 48 h. The percentage of EdU-incorporated cells was determined by quantifying the number of EdU-positive cells relative to the total number of nuclei detected per field.

*Western Blots.*

Cell lysates were prepared using lysis buffer (50 mM Tis-HCl, 150 mM NaCl, 1% NP40, 1 mM EDTA) with complete phosphatase and protease inhibitor cocktail (Roche, 4693159001). Primary antibodies were used at the following dilutions: mouse anti-Tfap4 (Abnova, H00007023-M01, 1:1,000), rabbit anti-α-SMA (CST, 19245S, 1:1,000), guinea pig anti-Vimentin (Progen, GP53, 1:2,000), rabbit anti-Fibronectin (Proteintech, 15613-1-AP, 1:1,000), rabbit anti-AKT (CST, 9272, 1:1,000), rabbit anti-pAKT (CST, 9271, 1:1,000), rabbit anti-Collagen I (Baijia, IPB0671, 1:1,000) and mouse anti-β-actin antibody (Santa Cruz Biotechnology, sc-47778, 1:1,000). Secondary antibodies including HRP-conjugated Affinipure Goat Anti-Mouse IgG (H+L), HRP-conjugated Affinipure Goat Anti-Rabbit IgG (H+L), HRP-conjugated Affinipure Goat Anti-Guinea pig IgG (H+L) were all from Proteintech. The target proteins were detected by chemiluminescence (EpiZyme). The blots were taken by ChemiDoc (Biorad) and the intensity of the target proteins was quantified with ImageJ software. The antibodies used in this assay were listed in Supplemental Table 5.

*Histology analysis.*

For immunofluorescence staining, mouse hearts were fixed with 0.5% PFA in DPBS supplemented with 5% sucrose overnight, followed by sequential dehydration with sucrose, and embedded in OCT compound for freezing in liquid nitrogen. Then the heart was dissected horizontally into 5 µm cryosections, which were stained with primary antibodies against Tfap4 (Abnova, H00007023-M01, 1:100) and Vimentin (Progen, GP53, 1:100), then with relative secondary antibodies (1:200) conjugated with Alexa 488 or Cy3 which were all from Jackson ImmunoResearch Inc. After washing, the nuclei were stained with Hoechst 33342 (Biosharp, BL803A). The sections were then imaged under Nikon microscope. The antibodies used in this assay were listed in Supplemental Table 5.

For Masson’s trichrome staining, the hearts were fixed with 4% PFA overnight and embedded in paraffin mold. The heart tissue was embedded in paraffin and dissected into 5 µm thick sections. Masson’s staining was performed using a modified Masson’s trichrome staining kit (BA4079B, BASO). Scar size was calculated as the percentage of total scar area divided by total left ventricle area. The results were analyzed using ImageJ in a double-blinded manner.

For wheat germ agglutinin (WGA) staining, the hearts were fixed with 4% PFA overnight and embedded in paraffin mold. The heart tissue was embedded in paraffin and dissected into 5 µm thick sections. In brief, slides were incubated with WGA conjugated to Alexa Fluor 555 (Invitrogen, W32464, 1:200) for 1 hour at room temperature and then washed with HBSS (Gibco, C14175500CP). To quantify the cross-sectional cell size, five independent hearts per group with fifty different views and positions were captured at 20× magnification. ImageJ was used to quantify the size of cardiomyocytes that were round and contained a nucleus. At least 500 cells per sample were quantified.

*Single‑cell transcriptomic datasets analysis.*

Data were downloaded from the Gene Expression Omnibus (GEO) database under the accession number GSE183852 and the gene count matrix was loaded into R (version 4.2.2) and pre-processed using Seurat (5.1.0), Single-Cell Pipeline (SCP) package (<https://github.com/zhanghao-njmu/SCP>), and Nebulosa package (<https://github.com/powellgenomicslab/Nebulosa>).

**References**

Wang, L., Yang, Y.C., Ma, H., Xie, Y.F., Xu, J., Near, D., Wang, H.F., Garbutt, T., Li, Y., Liu, J.D.*, et al.* (2022). Single-cell dual-omics reveals the transcriptomic and epigenomic diversity of cardiac non-myocytes. Cardiovascular Research *118*, 1548-1563.

**Supplemental Table 1 List of molecular cloning primers**

| **Symbol** | **Primer** | **Sequence** |
| --- | --- | --- |
| pLenti-*Tfap4* | Forward | TAGAGGATCCACCATGGAGTATTTCATGGTGCC |
|  | Reverse | GATTGTCGACTCAGGGAAGCTCCCCGTCCC |
| pHAGE-*Tfap4*-Flag | Forward | CGACGCGTGCCACCATGGAGTATTTCATGGTGCCCAC |
|  | Reverse | GATTGTCGACGGGAAGCTCCCCGTCCCCCAA |
| pGL3-Piezo2 | Forward | CGACGCGTTTTCATAGTGGATACCAGGGAG |
|  | Reverse | CCAAGCTTTCTTGTTTTCTCGGACTATTTTC |
| pGL3-Itga11 | Forward | CGACGCGTTCACCTGTCCTGGCAAAAAGAAGT |
|  | Reverse | CCAAGCTTAACCAAATCGGGGTCTCCTCTG |
| pGL3-△Piezo2 | Forward | TGGCTTTACCATACCTAATTTCCTCAAAATCCCTACTTTG |
|  | Reverse | ATTTTGAGGAAATTAGGTATGGTAAAGCCATAAAAGAAAC |
| pGL3-△Itga11 | Forward | CACCGCCCCAGCCTCCAGGGACTGCACCTGACGGTTCCGAG |
|  | Reverse | CAGGTGCAGTCCCTGGAGGCTGGGGCGGTGGAAACTGGAGG |

**Supplemental Table 2 List of shRNA oligos**

| **Genes** | **Forward sequence** | **Reverse sequence** |  |
| --- | --- | --- | --- |
| sh*Smad9*#1 | CCGGCGCATCCGAGTCACGTTTATACTCGAGTATAAACGTGACTCGGATGCGTTTTTG | AATTCAAAAACGCATCCGAGTCACGTTTATACTCGAGTATAAACGTGACTCGGATGCG |  |
| sh*Smad9*#2 | CCGGTGTTGCCTACTACGAACTAAACTCGAGTTTAGTTCGTAGTAGGCAACATTTTTG | AATTCAAAAATGTTGCCTACTACGAACTAAACTCGAGTTTAGTTCGTAGTAGGCAACA |  |
| sh*Tcf12*#1 | CCGGGAAGGCCTTGGCATCTATTTACTCGAGTAAATAGATGCCAAGGCCTTCTTTTTG | AATTCAAAAAGAAGGCCTTGGCATCTATTTACTCGAGTAAATAGATGCCAAGGCCTTC |  |
| sh*Tcf12*#2 | CCGGTGACGATTTCAACCGTGAATCCTCGAGGATTCACGGTTGAAATCGTCATTTTTG | AATTCAAAAATGACGATTTCAACCGTGAATCCTCGAGGATTCACGGTTGAAATCGTCA |  |
| sh*Mxi1*#1 | CCGGTCGGGAGTGACGAGGGTTATTCTCGAGAATAACCCTCGTCACTCCCGATTTTTG | AATTCAAAAATCGGGAGTGACGAGGGTTATTCTCGAGAATAACCCTCGTCACTCCCGA |  |
| sh*Mxi1*#2 | CCGGGCAGGATAATCAGGCATTAATCTCGAGATTAATGCCTGATTATCCTGCTTTTTG | AATTCAAAAAGCAGGATAATCAGGCATTAATCTCGAGATTAATGCCTGATTATCCTGC |  |
| sh*Bnc2*#1 | CCGGCGCTGACACTAACCTCTTATTCTCGAGAATAAGAGGTTAGTGTCAGCGTTTTTG | AATTCAAAAACGCTGACACTAACCTCTTATTCTCGAGAATAAGAGGTTAGTGTCAGCG |  |
| sh*Bnc2*#2 | CCGGGCCGAATGTTATTAGAATCAACTCGAGTTGATTCTAATAACATTCGGCTTTTTG | AATTCAAAAAGCCGAATGTTATTAGAATCAACTCGAGTTGATTCTAATAACATTCGGC |  |
| sh*Zfp82*#1 | CCGGCTTATGAGAAGCGCGCATTAACTCGAGTTAATGCGCGCTTCTCATAAGTTTTTG | AATTCAAAAACTTATGAGAAGCGCGCATTAACTCGAGTTAATGCGCGCTTCTCATAAG |  |
| sh*Zfp82*#2 | CCGGGCAAAGACTAAGGAAAGTAATCTCGAGATTACTTTCCTTAGTCTTTGCTTTTTG | AATTCAAAAAGCAAAGACTAAGGAAAGTAATCTCGAGATTACTTTCCTTAGTCTTTGC |  |
| sh*Vdr*#1 | CCGGCGTAAGTACAGGGAGCTATTCCTCGAGGAATAGCTCCCTGTACTTACGTTTTTG | AATTCAAAAACGTAAGTACAGGGAGCTATTCCTCGAGGAATAGCTCCCTGTACTTACG |  |
| sh*Vdr*#2 | CCGGCCTGAGATCAATCACATTTAACTCGAGTTAAATGTGATTGATCTCAGGTTTTTG | AATTCAAAAACCTGAGATCAATCACATTTAACTCGAGTTAAATGTGATTGATCTCAGG |  |
| sh*Rorb*#1 | CCGGGAAGGTTATTACAGCATAGATCTCGAGATCTATGCTGTAATAACCTTCTTTTTG | AATTCAAAAAGAAGGTTATTACAGCATAGATCTCGAGATCTATGCTGTAATAACCTTC |  |
| sh*Rorb*#2 | CCGGCGGGATAACAATGTCTGAGATCTCGAGATCTCAGACATTGTTATCCCGTTTTTG | AATTCAAAAACGGGATAACAATGTCTGAGATCTCGAGATCTCAGACATTGTTATCCCG |  |
| sh*Tfdp1*#1 | CCGGGCGTGTCTACGATGCCTTAAACTCGAGTTTAAGGCATCGTAGACACGCTTTTTG | AATTCAAAAAGCGTGTCTACGATGCCTTAAACTCGAGTTTAAGGCATCGTAGACACGC |  |
| sh*Tfdp1*#1 | CCGGCGATGCCTTAAATGTGCTAATCTCGAGATTAGCACATTTAAGGCATCGTTTTTG | AATTCAAAAACGATGCCTTAAATGTGCTAATCTCGAGATTAGCACATTTAAGGCATCG |  |
| sh*Eya2*#1 | CCGGCGGAGACTACAACACACACAACTCGAGTTGTGTGTGTTGTAGTCTCCGTTTTTG | AATTCAAAAACGGAGACTACAACACACACAACTCGAGTTGTGTGTGTTGTAGTCTCCG |  |
| sh*Eya2*#2 | CCGGCCATTTCAGAAGTGTCTTCTTCTCGAGAAGAAGACACTTCTGAAATGGTTTTTG | AATTCAAAAACCATTTCAGAAGTGTCTTCTTCTCGAGAAGAAGACACTTCTGAAATGG |  |
| sh*Foxc1*#1 | CCGGCCCTTCTATCGGGACAATAAGCTCGAGCTTATTGTCCCGATAGAAGGGTTTTTG | AATTCAAAAACCCTTCTATCGGGACAATAAGCTCGAGCTTATTGTCCCGATAGAAGGG |  |
| sh*Foxc1*#2 | CCGGGAACGGGAAAGTACCTGTTTACTCGAGTAAACAGGTACTTTCCCGTTCTTTTTG | AATTCAAAAAGAACGGGAAAGTACCTGTTTACTCGAGTAAACAGGTACTTTCCCGTTC |  |
| sh*Tshz3*#1 | CCGGCCCTTACATCACGCCAAATAACTCGAGTTATTTGGCGTGATGTAAGGGTTTTTG | AATTCAAAAACCCTTACATCACGCCAAATAACTCGAGTTATTTGGCGTGATGTAAGGG |  |
| sh*Tshz3*#2 | CCGGGCCTCTGTATAGTGTATATTTCTCGAGAAATATACACTATACAGAGGCTTTTTG | AATTCAAAAAGCCTCTGTATAGTGTATATTTCTCGAGAAATATACACTATACAGAGGC |  |
| sh*Cebpb*#1 | CCGGCTGACGCAACACACGTGTAACCTCGAGGTTACACGTGTGTTGCGTCAGTTTTTG | AATTCAAAAACTGACGCAACACACGTGTAACCTCGAGGTTACACGTGTGTTGCGTCAG |  |
| sh*Cebpb*#2 | CCGGCGCCTTTAGACCCATGGAAGTCTCGAGACTTCCATGGGTCTAAAGGCGTTTTTG | AATTCAAAAACGCCTTTAGACCCATGGAAGTCTCGAGACTTCCATGGGTCTAAAGGCG |  |
| sh*Kcnip3*#1 | CCGGGCCTATTCCTAGGTCAATAAACTCGAGTTTATTGACCTAGGAATAGGCTTTTTG | AATTCAAAAAGCCTATTCCTAGGTCAATAAACTCGAGTTTATTGACCTAGGAATAGGC |  |
| sh*Kcnip3*#2 | CCGGCCTGATGCGTTGCTGCTTAATCTCGAGATTAAGCAGCAACGCATCAGGTTTTTG | AATTCAAAAACCTGATGCGTTGCTGCTTAATCTCGAGATTAAGCAGCAACGCATCAGG |  |
| sh*Bhlhe40*#1 | CCGGAGAACGTGTCAGCACAATTAACTCGAGTTAATTGTGCTGACACGTTCTTTTTTG | AATTCAAAAAAGAACGTGTCAGCACAATTAACTCGAGTTAATTGTGCTGACACGTTCT |  |
| sh*Bhlhe40*#2 | CCGGGCGAGGTTACAGTGTTTATATCTCGAGATATAAACACTGTAACCTCGCTTTTTG | AATTCAAAAAGCGAGGTTACAGTGTTTATATCTCGAGATATAAACACTGTAACCTCGC |  |
| sh*Hoxb9*#1 | CCGGCAAAGAGAGGCCGGATCAAACCTCGAGGTTTGATCCGGCCTCTCTTTGTTTTTG | AATTCAAAAACAAAGAGAGGCCGGATCAAACCTCGAGGTTTGATCCGGCCTCTCTTTG |  |
| sh*Hoxb9*#2 | CCGGCAAAGAGAGGCCGGATCAAACCTCGAGGTTTGATCCGGCCTCTCTTTGTTTTTG | AATTCAAAAACAAAGAGAGGCCGGATCAAACCTCGAGGTTTGATCCGGCCTCTCTTTG |  |
| sh*Foxf2*#1 | CCGGGAGCGTGTGCCAAGATATTAACTCGAGTTAATATCTTGGCACACGCTCTTTTTG | AATTCAAAAAGAGCGTGTGCCAAGATATTAACTCGAGTTAATATCTTGGCACACGCTC |  |
| sh*Foxf2*#2 | CCGGGAGTTCTGCTCACCGATATTTCTCGAGAAATATCGGTGAGCAGAACTCTTTTTG | AATTCAAAAAGAGTTCTGCTCACCGATATTTCTCGAGAAATATCGGTGAGCAGAACTC |  |
| sh*Tfap4*#1 | CCGGACACAGCTCAAGCGCTTTATCCTCGAGGATAAAGCGCTTGAGCTGTGTTTTTTG | AATTCAAAAAACACAGCTCAAGCGCTTTATCCTCGAGGATAAAGCGCTTGAGCTGTGT |  |
| sh*Tfap4*#2 | CCGGCCTCCAGGGTTCCTGTTATTGCTCGAGCAATAACAGGAACCCTGGAGGTTTTTG | AATTCAAAAACCTCCAGGGTTCCTGTTATTGCTCGAGCAATAACAGGAACCCTGGAGG |  |
| sh*Tfap4*#3 | CCGGGGAACAGAGGCGAGCAGTTATCTCGAGATAACTGCTCGCCTCTGTTCCTTTTTG | AATTCAAAAAGGAACAGAGGCGAGCAGTTATCTCGAGATAACTGCTCGCCTCTGTTCC |  |
| sh*Tfap4*#4 | CCGGGGTGCCCTCTTTGCAACATTTCTCGAGAAATGTTGCAAAGAGGGCACCTTTTTG | AATTCAAAAAGGTGCCCTCTTTGCAACATTTCTCGAGAAATGTTGCAAAGAGGGCACC |  |
| sh*Piezo2*#1 | CCGGCCTCACAAAGAGCTACAATTACTCGAGTAATTGTAGCTCTTTGTGAGGTTTTTG | AATTCAAAAACCTCACAAAGAGCTACAATTACTCGAGTAATTGTAGCTCTTTGTGAGG |  |
| sh*Piezo2*#2 | CCGGGAGGACATTTACGCGCACATTCTCGAGAATGTGCGCGTAAATGTCCTCTTTTTG | AATTCAAAAAGAGGACATTTACGCGCACATTCTCGAGAATGTGCGCGTAAATGTCCTC |  |
| sh*Itga11*#1 | CCGGACGGCATTTGGCATTGAATTTCTCGAGAAATTCAATGCCAAATGCCGTTTTTTG | AATTCAAAAAACGGCATTTGGCATTGAATTTCTCGAGAAATTCAATGCCAAATGCCGT |  |
|  |  |  |  |
| sh*Itga11*#2 | CCGGCACACACACACACACACACAACTCGAGTTGTGTGTGTGTGTGTGTGTGTTTTTG | AATTCAAAAACACACACACACACACACACAACTCGAGTTGTGTGTGTGTGTGTGTGTG |  |
| sh*TFAP4*#1 | CCGGGGTGCCCTCTTTGCAACATTTCTCGAGAAATGTTGCAAAGAGGGCACCTTTTTG | AATTCAAAAAGGTGCCCTCTTTGCAACATTTCTCGAGAAATGTTGCAAAGAGGGCACC |  |
| sh*TFAP4*#2 | CCGGCGATGTCATTTGGGTCTCTTTCTCGAGAAAGAGACCCAAATGACATCGTTTTTG | AATTCAAAAACGATGTCATTTGGGTCTCTTTCTCGAGAAAGAGACCCAAATGACATCG |  |
| sh*TFAP4*#3 | CCGGCCTCGGTCATCAACTCTGTTTCTCGAGAAACAGAGTTGATGACCGAGGTTTTTG | AATTCAAAAACCTCGGTCATCAACTCTGTTTCTCGAGAAACAGAGTTGATGACCGAGG |  |
| shTFAP4#4 | CCGGGCCTTGCCAACATTCCACTAACTCGAGTTAGTGGAATGTTGGCAAGGCTTTTTG | AATTCAAAAAGCCTTGCCAACATTCCACTAACTCGAGTTAGTGGAATGTTGGCAAGGC |  |

**Supplemental Table 3 List of qPCR primers**

| **Genes** | **Taxon** | **Forward primer** | **Reverse primer** |
| --- | --- | --- | --- |
| Smad9 | Mouse | CGGGTCAGCCTAGCAAGTG | GAGCCGAACGGGAACTCAC |
| Tcf12 | Mouse | ATGTACTGTGCTTATCCTGTCCC | GGTGCATATACCGTTTTCCCATT |
| Mxi1 | Mouse | AACATGGCTACGCCTCATCG | CGGTTCTTTTCCAACTCATTGTG |
| Bnc2 | Mouse | GCTGGCAAGGTACTGGACC | CCACGATGGATTTGGTTTCCC |
| Zfp82 | Mouse | AGCCAGATGTGATTTCCTTACTG | GGTTGGTCTCATCCTTAGTTTCC |
| Vdr | Mouse | ACCCTGGTGACTTTGACCG | GGCAATCTCCATTGAAGGGG |
| Rorb | Mouse | GCAGCATTAGCAATGGCCTC | GACGGCTGACCGGAATCTATG |
| Tfdp1 | Mouse | TTGAAGCCAACGGAGAACTAAAG | TGGACTGTCCGAAGGTTTTTG |
| Eya2 | Mouse | GCTGACACACACCTGTTCTTC | CTATCAAGCCACCCACGTTGT |
| Foxc1 | Mouse | CCCCGGACAAGAAGATCACTC | AGGTTGTGCCGTATGCTGTTC |
| Tshz3 | Mouse | GCAGCAGCCTATGTTTCCGAT | ACTGATGTGAGACTCGCTGTC |
| Cebpb | Mouse | CAAGAAGACGGTGGACAAGC | AGCTGCTCCACCTTCTTCTG |
| Kcnip3 | Mouse | TGAAGGCATCAGATGGCAACC | CCGTGGATAACTCCAGTTCACT |
| Bhlhe40 | Mouse | ACGGAGACCTGTCAGGGATG | GGCAGTTTGTAAGTTTCCTTGC |
| Hoxb9 | Mouse | GCACGCCCGAGTACAGTTT | GGCAGAGGGGTTGGTTTGA |
| Foxf2 | Mouse | CGTCCTCTTCTAACTCCGTCA | ATGTACGAGTAAGGAGGCTTCT |
| Tfap4 | Mouse | GCTCTGTAGCCTAGCCAACAT | GAAGCCCGCGTTGATACTCT |
| Fn1 | Mouse | ATGTGGACCCCTCCTGATAGT | GCCCAGTGATTTCAGCAAAGG |
| Col8a1 | Mouse | ACTCTGTCAGACTCATTCAGGC | CAAAGGCATGTGAGGGACTTG |
| IL6 | Mouse | TAGTCCTTCCTACCCCAATTTCC | TTGGTCCTTAGCCACTCCTTC |
| IL6R | Mouse | CCTGAGACTCAAGCAGAAATGG | AGAAGGAAGGTCGGCTTCAGT |
| Mmp2 | Mouse | CAAGTTCCCCGGCGATGTC | TTCTGGTCAAGGTCACCTGTC |
| Mmp9 | Mouse | CTGGACAGCCAGACACTAAAG | CTCGCGGCAAGTCTTCAGAG |
| Mmp14 | Mouse | CAGTATGGCTACCTACCTCCAG | GCCTTGCCTGTCACTTGTAAA |
| Timp1 | Mouse | CGAGACCACCTTATACCAGCG | ATGACTGGGGTGTAGGCGTA |
| Col14a1 | Mouse | TTTGGCGGCTGCTTGTTTC | CGCTTTTGTTGCAGTGTTCTG |
| Acta2 | Mouse | GTCCCAGACATCAGGGAGTAA | TCGGATACTTCAGCGTCAGGA |
| Ctgf | Mouse | GGGCCTCTTCTGCGATTTC | ATCCAGGCAAGTGCATTGGTA |
| Wisp2 | Mouse | CGCTGTGATGACGGTGGTTT | CCTGGCACCTGTATTCTCCTG |
| Postn | Mouse | CCTGCCCTTATATGCTCTGCT | AAACATGGTCAATAGGCATCACT |
| Aoc3 | Mouse | GAAGACCACCCTAGTGCTCCT | ATGAAGAGGTTGGCTCAGTCC |
| Has2 | Mouse | TGTGAGAGGTTTCTATGTGTCCT | ACCGTACAGTCCAAATGAGAAGT |
| Col6a6 | Mouse | GTGGACAGCTCCGATCACCTA | GGGCGTTCCCTATCTTCAGG |
| Fgf7 | Mouse | CTCTACAGGTCATGCTTCCACC | ACAGAACAGTCTTCTCACCCT |
| Fgf9 | Mouse | ATGGCTCCCTTAGGTGAAGTT | TCATTTAGCAACACCGGACTG |
| Fgf13 | Mouse | CTCATCCGGCAAAAGAGACAA | TTGGAGCCAAAGAGTTTGACC |
| Cd93 | Mouse | ATCTCAACTGGTTTGTTCCTGC | ACTCTTCACGGTGGCAAGATT |
| Sfrp1 | Mouse | CAACGTGGGCTACAAGAAGAT | GGCCAGTAGAAGCCGAAGAAC |
| Gdnf | Mouse | TCCAACTGGGGGTCTACGG | GCCACGACATCCCATAACTTCAT |
| Osr1 | Mouse | TACTCTTTCCTTCAGGCAGTGA | GATCGAGGCAAGTGCATGG |
| Kit | Mouse | GCCACGTCTCAGCCATCTG | GTCGCCAGCTTCAACTATTAACT |
| Ptprz1 | Mouse | GGAGAAGAACAGAACATCGTCC | TCATTGCTCTGGTAATAGCCCA |
| Mcam | Mouse | CCCAAACTGGTGTGCGTCTT | GGAAAATCAGTATCTGCCTCTCC |
| Itga11 | Mouse | TGCCCCAATGGAAACCAATG | CACTCGTGCGACCAGAGAG |
| Piezo2 | Mouse | AATCAAACCAACATTCCCCTTCA | CAGGTAGACGAGCAAAGGAGA |
| Piezo1 | Mouse | AGGACTTCCCCACCTATTGG | CCAGGGATGAGGATACTGGAAAA |
| TFAP4 | Human | GGCAAAATCTGGACACCATCGTG | GACAGGCTTCACGATGACAGCT |
| ACTA2 | Human | CTATGCCTCTGGACGCACAACT | CAGATCCAGACGCATGATGGCA |
| COL1A1 | Human | GATTCCCTGGACCTAAAGGTGC | AGCCTCTCCATCTTTGCCAGCA |
| FN1 | Human | TTCACGTCTGTCACTTCCACA | TCTCATTCAACAAGAAACCACTG |
| MMP2 | Human | CCTGCGACCAACTCCACGTCT | TTCACCTCACAGCTGCTGTCGT |
| PIEZO2 | Human | GACGGACACAACTTTGAGCCTG | CTGGCTTTGTTGGGCACTCATTG |
| ITGA11 | Human | GCCTCCAGTATTTTGGCTGCAG | GCTCAAAGTGGAGGCTGGCATT |
| MMP9 | Human | GCCACTACTGTGCCTTTGAGTC | CCCTCAGAGAATCGCCAGTACT |

**Supplemental Table 4 List of ChIP-qPCR primers**

|  | **Forward primer** | **Reverse primer** |
| --- | --- | --- |
| Itga11-1 | TCAAACTGTTCCCAGCATGCAC | GGGATGAGGCTGACACTGCTT |
| Itga11-2 | ACTCTGATGCCCTACCCACT | GGAGGTAGGCATTCCATGACA |
| Itga11-3 | AGGGTTTGTGGTTTGTGGTGA | CAGCTGTTCCCTTGCATGTTG |
| Piezo2-1 | CCCTCGGTTGCAAGTTTAGCAC | TGAAAGCGCCATTCACCAGTC |
| Piezo2-2 | TCTCAAATGAGTCCAGGGGGC | GGCCCACGTTTTACTCTGCT |
| Piezo2-3 | GACTCCACTTGCATGGCGTT | TACTTTCTTGGCGGCTGGGA |

**Supplemental Table 5. List of antibodies**

**Primary antibodies**

| **Antibodies** | **Usage (dilution)** | **Source** | **Identifier** |
| --- | --- | --- | --- |
| anti-Ki67 antibody | ICC (1:250) | Abcam | ab16667 |
| anti-Vimentin antibody | ICC (1:200) | Progen | GP53 |
|  | IF (1:100) |  |  |
| F-Actin/ phalloidin-TRITC | ICC (1:500) | Solarbio | CA1610 |
| anti-Paxillin antibody | ICC (1:500) | Abcam | ab32084 |
| anti-α-SMA antibody | WB (1:1000) | Cell Signaling Technology | 9271 |
| anti- AKT antibody | WB (1:1000) | Cell Signaling Technology | 9272 |
| Phospho-Akt (Ser473) Antibody | WB (1:1000) | Cell Signaling Technology | 9271 |
| anti-Collagen I | WB (1:1000) | Baijia | IPB0671 |
| anti-Flag antibody | ChIP (5 μg per sample) | Sigma | F3165 |
| normal mouse IgG | ChIP (5 μg per sample) | Santa Cruz Biotechnology | B0619 |
| anti-Fibronectin | ICC (1:200) | Proteintech | 15613-1-AP |
|  | WB (1:1000) |  |  |
| anti-Tfap4 antibody | WB (1:1000) | Abnova | H00007023-M01 |
|  | IF (1:100) |  |  |
| β-Actin Antibody (C4) | WB (1:1000) | Santa Cruz Biotechnology | sc-47778 |
| Wheat germ agglutinin | IF (1:200) | Invitrogen | W32464 |

**Secondary antibodies**

| **Antibodies** | **Usage (dilution)** | **Source** | **Identifier** |
| --- | --- | --- | --- |
| Alexa Fluor® 488 affiniPure donkey anti-mouse IgG (H+L) | IHC (1:200) | Jackson ImmunoResearch Inc | 715-545-150 |
| Cy3 AffiniPure Donkey Anti-guinea pig IgG (H+L) | IHC (1:200) | Jackson ImmunoResearch Inc | 706-165-148 |
| Alexa Fluor® 488 affiniPure donkey anti-rabbit IgG (H+L) | ICC (1:500) | Jackson ImmunoResearch Inc | 711-545-152 |
| Cy™3 affinipure donkey anti-rabbit IgG (H+L) | ICC (1:500) | Jackson ImmunoResearch Inc | 711-165-152 |
| HRP-conjugated Goat Anti-Guinea pig IgG(H+L) | WB (1:5000) | Proteintech | SA00001-12 |
| Goat Anti-Rabbit IgG, Peroxidase Conjugated, H+L | WB (1:5000) | Biosharp | BL003A |
| HRP-conjugated Goat Anti-Mouse IgG(H+L) | WB (1:5000) | Proteintech | SA00001-1 |
